# Promyelocytic leukemia protein deficiency leads to spontaneous formation of liver tumors in hepatitis C virus transgenic mice

**DOI:** 10.1002/cam4.2162

**Published:** 2019-05-29

**Authors:** Katja Straub, Peri Husen, Hideo A. Baba, Martin Trippler, Heiner Wedemeyer, Kerstin Herzer

**Affiliations:** ^1^ Department of Gastroenterology and Hepatology, Faculty of Medicine, University Hospital Essen University of Duisburg‐Essen Essen Germany; ^2^ Department of General‐, Visceral‐ and Transplantation Surgery, Faculty of Medicine, University Hospital Essen University of Duisburg‐Essen Essen Germany; ^3^ Institute of Pathology, Faculty of Medicine, University Hospital Essen University of Duisburg‐Essen Essen Germany

**Keywords:** hepatitis C virus, hepatocellular carcinoma, liver tumor, promyelocytic leukemia protein

## Abstract

Persistent infection with hepatitis C virus (HCV) is a known risk factor for the development of hepatocellular carcinoma (HCC). The lack of the tumor suppressor promyelocytic leukemia protein (PML) in combination with HCV fosters hepatocarcinogenesis via induction of HCC using diethylnitrosamine (DEN) in a rodent model. However, the spontaneous development of malignant lesions in PML‐deficient mice with an HCV‐transgene (HCV_tg_) has not been investigated thus far. We crossed PML‐deficient mice with HCV transgene expressing mice and observed the animals for a period of 12 months. Livers were examined macroscopically and histologically. Gene expression analysis was performed on these samples, and compared with expression of selected genes in human samples of patients undergoing liver transplantation for HCC. In vitro studies were performed in order to analyze the selected pathways. Genetic depletion of PML in combination with HCV_tg_ coincided with an increased hepatocyte proliferation, resulting in development of HCCs in 40% of the PML‐deficient livers. No tumor development was observed in mice with either the PML‐knockout (PML^−/−^) or HCV_tg_ alone. Gene expression profiling uncovered pathways involved in cell proliferation, such as NLRP12 and RASFF6. These findings were verified in samples from human livers of patients undergoing liver transplantation for HCC. Further in vitro studies confirmed that lack of PML, NLRP12, and RASFF6 leads to increased cell proliferation. The lack of PML in combination with HCV is associated with increased cell proliferation, fostering tumor development in the liver. Our data demonstrate that PML acts as an important tumor suppressor in HCV‐dependent liver pathology.

## 1 INTRODUCTION

Liver cancer has been identified as a growing clinical problem, being the second most common cause of cancer‐related deaths globally, and estimated to be responsible for nearly 9.1% of deaths in 2012 (GLOBOCAN, http://globocan.iarc.fr). Hepatocellular carcinoma (HCC) represents about 80%‐90% of all primary liver cancers, correlating with a high mortality and very poor prognosis.^1^ Clinical risk factors for developing HCC have long been identified, and include alcohol intake, nonalcoholic steatohepatitis, the fungal metabolite aflatoxin B1, as well as hepatitis B (HBV), and C (HCV) virus infection. The World Health Organization (WHO) estimates that about 71 million people are chronic carriers of the hepatotropic hepatitis C virus (HCV; WHO, www.who.int), with HCV being the leading cause of HCC and the first indication for liver transplantation for patients in developed countries.^2^, ^3^, ^4^ The risk of developing HCC correlates with fibrosis stage, with an increasing incidence once cirrhosis has developed (1%‐7% per year).^5^, ^6^ HCV‐related HCC development is a multi‐step process that takes place over decades. Even after successful treatment of HCV, a high risk of HCC development persists.

Two mechanistic components have been identified that foster HCV‐associated hepatocarcinogenesis: First, an inflammatory stage induced by chronic hepatocyte damage leading to the release of reactive oxygen species, apoptosis signals, nucleotides, and hedgehog ligands,^7^ ultimately leading to the activation of hepatic stellate cells and the progression of fibrosis toward cirrhosis. Second, a direct effect of HCV core as well as nonstructural proteins on profibrogenic genes such as TGF‐beta,^8^ as well as intracellular signaling pathways, including the mitogen‐activated protein kinase cascade.^9^ Several viral proteins have been identified in liver carcinogenesis, with HCV core protein being the most prevalent regarding cellular transformation and direct interaction with the tumor suppressor protein p53.^10^, ^11^

Despite our growing knowledge of HCV interactions, HCV‐mediated transformation and the progression to hepatocellular cancer remains incompletely understood.

We previously uncovered a so far unidentified connection between HCV core and promyelocytic leukemia‐nuclear bodies (PML‐NBs).^12^, ^13^ PML is a tumor suppressor protein, initially described in its role during the pathogenesis of acute promyelocytic leukemia (APL).^14^ PML localizes to nuclear bodies (NBs), which are multi‐protein complexes. As of today, loss of PML has been described in numerous human cancers.^15^ In our in vivo approach, we could show that the deficiency of PML leads to increased HCC development in HCV‐transgenic mice when animals were treated with an established protocol of phenobarbital and diethylnitrosamine (DEN).^13^ Of note, HCV core protein targets PML‐NBs and inactivates the tumor suppressor function of PML through interference with the apoptosis‐induction of PML‐isoform IV in human hepatoma cells.^12^

We have now investigated these mice for spontaneous development of dysplastic nodules as well as HCC. We could show that by the age of 12 months, 40% of all mice of the HCV‐transgenic, PML‐deficient group (HCV_tg_PML^−/−^) developed liver tumors, when compared to mice that were just PML^−/− ^(but HCV negative), or HCV_tg _(but PML wild type), or wild type animals. Our in vivo data demonstrate a direct effect of HCV toward spontaneous liver tumor development under PML deficiency. This highlights the importance and direct correlation of PML as a tumor suppressor during HCV‐related carcinogenesis. Importantly, we have also reflected results of gene expression profiling from these mice with a human cohort of patients that were liver transplanted for HCC.

## 2 MATERIALS AND METHODS

### 2.1 Animals and genotyping

PML^−/−^ mice (within a 129Sv genetic background) were generated by Pier Paolo Pandolfi (Beth Israel Diaconess Medical Center) as described previously.^16^ HCV transgenic FL‐N/35 mice, carrying the full‐length protein‐coding region of HCV genotype 1b, (within a C3H/C57BL6 genetic background) were generated by Herve Lerat (INSERM) and Stanley M. Lemon (UTMB).^17^ The two mouse stains were crossed, and the following four genotypes were used for this study: (a) WT; (b) PML^−/−^; (c) HCV_tg_; (d) PML^−/−^HCV_tg_. Genotyping of all used genotypes was performed by PCR and analyzed on 2% agarose gels. For a complete list of primer sequences see Table S1.

Transgenic mice (PML^−/−^, HCV_tg_, PML^−/−^HCV_tg_) as well as WT mice were left untreated under frequent observation. After 1 year 10 male mice of each group were sacrificed and analyzed. The general habitus as well as bodyweight were determined. After complete necropsy, the liver tissue of all animals was macrodissected and processed according to the analyses described below.

### 2.2 Patients

Liver tissue of patients undergoing liver transplantation was collected and further processed for RNA extraction (for indications see Table S2) as described elsewhere,^18^ and liver tissue of patients undergoing liver resection was collected for protein analysis (for indications see Table S3). Patients had given informed consent on scientific use of resected liver tissue, and all human tissue samples were collected in accordance with the Declaration of Helsinki. Samples for further analysis were collected from HCC tissue (TT) and tumor‐surrounding liver tissue (TST), as well as from livers without evidence of hepatocellular carcinoma formation (NTT) of each patient's explanted liver at time of transplantation. The underlying liver disease of these patients is listed in Tables S2 and S3.

### 2.3 Histology and immunohistochemical analysis

Liver samples were formalin‐fixed, paraffin‐embedded, sectioned at 4 µm, and processed routinely for H&E staining. Immunohistochemical staining of glutamine synthetase (ab49873; 1:10 000; Abcam) and Ki67 (ab66155; 1:1000; Abcam) was performed on formalin‐fixed, paraffin‐embedded liver sections with the Histar Detection Kit Star3000a according to the manufacturer's instructions (Bio‐Rad Laboratories, Inc). Morphometry was used to quantify the stained tissue area using ImageJ software (National Institute of Health). Slides were counterstained with hematoxylin.

### 2.4 Western blot

For protein isolation, 30 mg of human liver or tumor tissue were macrodissected and lysed in ice‐cold RIPA buffer (50 mmol/L Tris, 150 mmol/L NaCl, 1% NP‐40, 0.5% sodiumdeoxycholate, 1 mmol/L EDTA, pH 7.4) complemented with protease and phosphatase inhibitor (Halt Phosphatase Inhibitor Single‐Use Cocktail, Thermo Scientific) by mechanic homogenization using TissueRuptor (Qiagen Inc). Protein lysate was incubated on ice for 30 minutes, and cell debris was removed through 20 minutes of centrifugation at 12 000 × *g* and 4°C. The supernatant was collected and total protein concentration was determined using the Pierce™ BCA Protein Assay Kit (Thermo Fischer Scientific).

Cultured cells were washed in ice‐cold PBS and lysed by adding 100 µL RIPA buffer (50 mmol/L Tris, 150 mmol/L NaCl, 1% NP‐40, 0.5% Sodium Deoxycholate, 1 mmol/L EDTA, pH 7.4) per well of a 12‐well plate. Cell lysates were cleaned by 10 minutes of centrifugation at 5000 *g* and 4°C. Total protein concentration was determined using the Pierce™ BCA Protein Assay Kit (Thermo Fischer Scientific).

For Western blots, 25 µg of total protein lysate was separated by SDS gel electrophoresis and transferred to PVDF‐membranes. For protein detection, the following antibodies were used: rabbit anti‐PML (H‐238; 1:1000; Santa Cruz Biotechnology, Inc); rabbit anti‐NLRP12 (ab93113; 1:200; Abcam); rabbit anti‐RASSF6 (ab220111; 1:200; Abcam); rabbit anti‐GAPDH (14C10; 1:1000; Cell Signaling Technology); anti‐rabbit mouse Antibody (31458; 1:10.000; Thermo Fisher Scientific).

### 2.5 Reverse transcription and real‐time quantitative PCR

For RNA isolation, 30 mg of murine or human liver or tumor tissue was lysed in QIAzol Lysis Reagent (Qiagen Inc) by mechanic homogenization using TissueRuptor (Qiagen Inc). Total RNA and miRNA were isolated by phenol‐chloroform extraction followed by isopropanol precipitation. RNA purification was done using the miRNeasy kit (Qiagen Inc) according to the manufacturer's instructions.

Cultured cells were lysed in 350 µL RLT buffer (mRNeasy kit [Qiagen Inc]) and RNA isolation was performed according to the manufacturer's instructions.

First strand cDNA synthesis was performed with 1 µg total RNA using M‐MLV reverse transcriptase (Thermo Fisher Scientific Inc) and 6 µM Random Primer Mix (New England Biolabs) according to the manufacturer's instructions. Quantitative rt‐PCR was performed with the QuantiFast SYBR green PCR Kit (Qiagen Inc) on the C1000 Touch Thermal Cycler (Bio‐Rad Laboratories, Inc). Gene expression analysis was performed with Microsoft Excel (Microsoft Corp.) and GraphPad Prism6 (GraphPad Software, Inc) software after normalization to β‐Actin (ACTB) in murine and cell culture samples and glyceraldehyde‐3‐phosphate dehydrogenase (GAPDH) in human samples. The sequences of all primers are listed in Table S1.

### 2.6 Gene expression profiling by microarray

For whole genome expression analysis on the GeneChip® HT MG‐430 PM Array Plate (Affymetrix Inc), total RNA was transcribed with 3′ IVT Express Kit (Affymetrix Inc) according to the manufacturer's instruction and further processed with GeneTitanTM Hybridization, Wash, and Stain Kit for 3' IVT Arrays (Affymetrix Inc) and the GeneTitan® Wash Buffers A and B Module (Affymetrix Inc). The experiments were performed on the GeneTitan® Instrument (Affymetrix Inc). The data discussed in this manuscript have been deposited in NCBI's Gene Expression Omnibus and are accessible through GEO Series accession number GSE119806 (https://www.ncbi.nlm.nih.gov/geo/query/acc.cgi?acc=GSE119806).

### 2.7 Hierarchical cluster analysis

Background signal was excluded by setting the general expression value in WT samples >200. Using two‐sided *t* Test between WT and NTT as well as NTT and TST, genes showing significantly different expression levels were identified. Hierarchical cluster analysis was performed with relative expression values normalized to WT samples using the Multiple Experiment Viewer (MeV 4.9.0). We performed average linkage clustering by genes and samples.

### 2.8 Gene set enrichment analysis

For gene set enrichment analysis (GSEA) 3.0 (JAVA version) provided by http://software.broadinstitute.org/gsea/index.jsp was used. Gene sets for liver cancer signatures as well as biocarta and KEGG were used as reference. The GSEA was performed with 1000 permutations and a limited number of genes between 4 and 500. The statistics of GSEA includes Normalized Enrichment Score (NES), False Discovery Rate (FDR) and *P*‐value. The results are displayed as heatmap.

### 2.9 Cell culture

Huh7 and HepG2 cells were obtained from the American Type Culture Collection (ATCC). Both cell lines were cultured in Dulbecco's modified essential medium (Gibco) supplemented with 10% heat‐inactivated fetal bovine serum (Gibco) and 100 U/mL penicillin and 100 µg/mL streptomycin sulfate (Gibco) in a humidified incubator with 5% CO_2_ at 37°C.

For protein analysis, 1 × 10^5^ Huh7 or HepG2 cells were seeded per well of a 12‐well plate. For RNA analysis, 5 × 10^4^ Huh7 or HepG2 cells were seeded per well of a 24‐well plate. For proliferation assays, 1 × 10^4^ Huh7 or HepG2 cells were seeded per well of a 96‐well plate.

All cells were transfected with 20 nmol/L control or gene‐specific si‐RNA (siCtrl, SI03650318; si*Pml*, SI00034664, si*Rassf6*, SI03243275, and SI04323991; si*Nlrp12*, SI04272758, and SI04146933; Qiagen Inc). Transfection was performed with the siLentFect™ Lipid Reagent according to the manufacturer's instructions (Bio‐Rad Laboratories, Inc). After 72 hours, cells were harvested according to the requirements of the subsequent analysis.

For cell proliferation analysis, cells were incubated with BrdU for 2 hours and analyzed by BrdU incorporation assay (Roche, Basel, Switzerland) according to the manufacturer's instruction and measured by luminescence detection. Fold change of the fluorescence signal of gene‐specific siRNA transfected cells was measured and normalized to cells transfected with siCtrl. Cell proliferation assay was performed in triplicates of at least three independent analyses.

### 2.10 Bioinformatics and statistical analysis

Fold change of gene and protein expression as well as cell proliferation is provided as median and range of at least three independent experiments when not stated otherwise. The comparison of two groups was performed by student's *t *test. Three or more groups were compared by two‐way ANOVA. A *P*‐value < 0.05 was considered statistically significant.

## 3 RESULTS

### 3.1 PML‐deficiency leads to spontaneous development of HCC under the presence of HCV‐transgene in vivo

**Figure 1:**
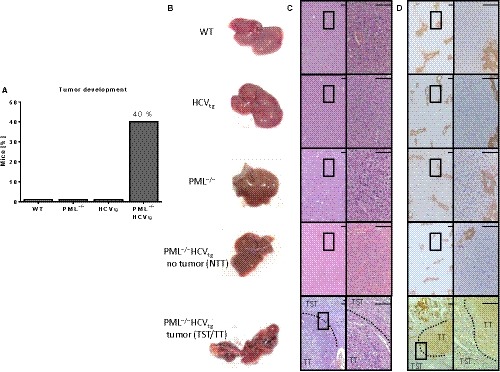
PML deficiency leads to spontaneous development of hepatocellular carcinoma (HCC) under the presence of HCV‐transgene in vivo. A, Graph shows percentage of mice of each group that developed a macroscopic liver tumor within the observation period of 12 month. Ten animals were used in each experimental group. B, Macroscopic images of livers from each experimental group confirm spontaneous tumor development in PML^−/−^HCV_tg_ animals. Sixty percent of PML^−/−^HCV_tg_ mice did not develop liver tumors (nontumorous tissue, NTT), whereas 40% of these mice developed HCC (tumorous tissue, TT; tumor‐surrounding tissue, TST). C, H&E staining of paraffin‐embedded liver sections of each experimental group. D, Glutamine synthase staining confirms high expression within HCC (TT) in PML^−/−^HCV_tg_ animals, compared to none within the tumor‐surrounding tissue (TST) and nontumorous livers (NTT) of PML^−/−^HCV_tg_ mice. N = 10 mice were analyzed per experimental group. Dashed border lines show the intersection between HCC (TT) and tumor‐surrounding liver tissue (TST). Scale bar equals 100 µm. Higher magnification of squared areas (4× magnification) is presented to the right of each image (20× magnification). NTT, nontumorous tissue; TST, tumor‐surrounding tissue; TT, tumor tissue

In order to investigate the contribution of PML and HCV to liver tumor development, we selected mice that either carried the HCV transgene (HCV_tg_), were PML‐deficient (PML^−/−^) or had a combined genotype of PML knockout and HCV transgene (PML^−/−^HCV_tg_) and compared these groups to wild‐type mice (WT; Figure S1A,B). Mice were observed until they reached the age of 12 months, after which we assessed the presence of macroscopic liver tumors at the surface of each animal's liver in each experimental group. Within the PML^−/−^HCV_tg _group, 40% of all animals presented with at least one macroscopic tumor by the age of 12 months, whereas there were no lesions present in livers of mice of the three other groups (HCV_tg_, PML or WT; Figure 1A). H&E staining showed no overall fibrotic changes within each liver of each experimental group (Figure 1B). Immunohistochemistry confirmed high expression of glutamine synthetase within the liver cell tumors of the PML^−/−^HCV_tg _group (Figure 1D), as typically observed in HCC tissue.

### 3.2 PML‐deficiency leads to increased proliferation of hepatocytes under presence of HCV‐transgene in vivo

**Figure 2:**
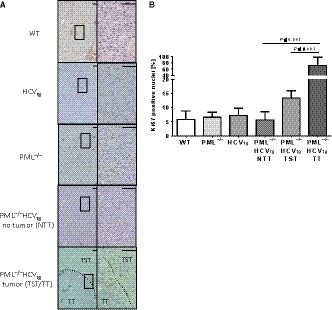
PML deficiency leads to increased proliferation in hepatocytes under the presence of HCV‐transgene in vivo. A, Ki67 staining was performed on paraffin‐embedded liver sections. Within the PML^−/−^HCV_tg _group, some animals did not develop liver tumors (NTT), whereas 40% of this group presented with liver tumors. In the latter, tumorous tissue (TT) is depicted with tumor‐surrounding tissue (TST). B, Quantification of Ki67‐positive nuclei for each experimental group. Five independent fields (40×) of n = 5 mice per genotype were quantified for Ki67‐positive hepatocytes. Tumorous tissue, as well as tumor‐surrounding tissue of the PML^−/−^HCV_tg_ showed the highest rate of tumor development compared to all other groups. N = 10 mice were analyzed per experimental group. Scale bar equals 100 µm. Higher magnification of squared areas (4× magnification) is presented to the right of each image (20× magnification). NTT, nontumorous tissue; TST, tumor‐surrounding tissue; TT, tumor tissue

Since mice with a genetic combination of PML‐deficiency and presence of HCV‐transgene exhibit increased expression of glutamine synthetase as well as macroscopic liver tumors, we assessed the proliferation of hepatocytes in each experimental group using Ki67 immunohistochemistry (Figure 2A,B). Mice of the WT, PML^−/−^, and HCV_tg _group exhibited <10% of Ki67‐positive nuclei per field. When the PML^−/−^HCV_tg_ group was analyzed, mice that did not develop liver tumors also showed <10% of Ki67‐positive nuclei. However, when liver tissue of PML^−/−^HCV_tg_ mice that did develop liver tumors was assessed, there was a significant increase of Ki67‐positive nuclei of up to 14% in the tumor‐surrounding tissue (TST) and up to 60% in the tumor tissue (TT) itself when compared to all other experimental groups (Figure 2B). This indicates that a subgroup (40%) of PML^−/−^HCV_tg _animals that did develop liver tumors harbor a permissive environment that facilitates tumor growth.

### 3.3 PML‐deficiency in combination with HCV leads to decreased expression of genes associated with tumor‐suppression

**Figure 3:**
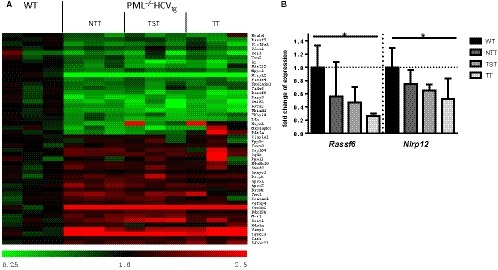
PML deficiency in combination with HCV leads to decreased expression of genes associated with tumor‐suppression. A, Heat map of gene expression profiling depicts upregulation of genes associated with cell proliferation and downregulation of tumor‐suppressor genes in PML^−/−^HCV_tg_ mice. N = 3 mice are represented for each experimental group. B, Whole liver mRNA expression of *Rassf6*, *Nlrp12* confirms increased expression within nontumorous livers (NTT), as well as tumor‐surrounding tissue (TST) and tumorous tissue (TT) of PML^−/−^HCV_tg_ animals compared to wild type animals. Bars represent the mean of n = 10 animals per experimental group (WT n = 10, PML^−/−^HCV_tg_ n = 10). Out of the PML^−/−^HCV_tg_ group, mice were divided as per presence of tumors (NTT n = 6, TST and TT n = 4). mRNA is expressed normalized to *Gapdh* (*P < 0.05; error bars indicate SEM). WT, wild type; NTT, nontumorous tissue; TST, tumor‐surrounding tissue; TT, tumor tissue

We next aimed at identifying mechanisms that may contribute to the increased proliferation of hepatocytes in a subgroup of PML^−/−^HCV_tg _mice that did develop tumors vs the subgroup of PML^−/−^HCV_tg _mice that never developed lesions within 12 months, as well as WT animals. We performed gene expression profiling of whole liver mRNA from WT animals, as well as from whole liver of PML^−/−^HCV_tg _mice that never developed a liver tumor (NTT), and compared these with gene expression profiling of whole liver mRNA from tumorous tissue (TT) and tumor surrounding tissue (TST) from PML^−/−^HCV_tg _mice that did develop liver masses (Figure 3A, Table S4). GSEA identified multiple signaling pathways altered between WT and NTT, NTT and TST as well as TST and TT tissue, in particular gene sets related to liver tumor development (Table S5, Figure S2).

We next analyzed the gene expression data regarding genes showing a continuous and stepwise alteration of expression from WT through NTT to TST and TT. Here we identified two genes, RAS‐Association Domain Family 6 (*Rassf6)* and NOD‐like receptor *Nlrp12*, which are known to be associated with PML and NFκB signaling pathway. *Rassf6* and *Nlrp12* were significantly downregulated in TT of PML^−/−^HCV_tg _mice vs WT animals (Figure 3B). Of note, a stepwise decrease in expression of these genes was observed when comparing WT animals with PML^−/−^HCV_tg _mice that did not develop liver tumors (NTT), as well as TST of PML^−/−^HCV_tg _mice that did develop liver tumors.

### 3.4 Tumor tissue of patients undergoing liver transplantation and liver resection for HCC presents with decreased expression of PML, RASSF6, and NLRP12 in vivo

**Figure 4:**
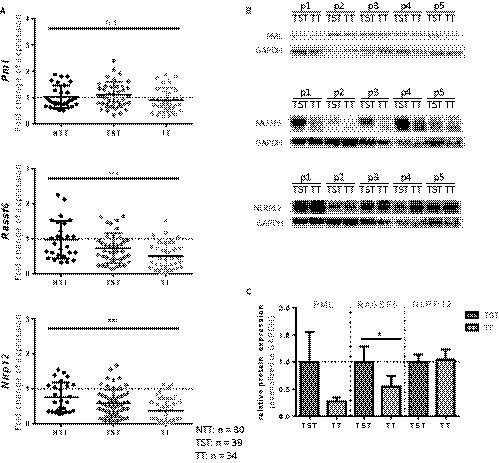
Tumor tissue of patients undergoing liver transplantation or liver resection for hepatocellular carcinoma (HCC) presents with decreased expression of PML, RASSF6, and NLRP12 in vivo. A, Whole liver mRNA expression of* PML*, *RASSF6,* and *NLRP12* at time of liver resection confirms decreased expression of these genes in tumor tissue when compared with nontumorous tissue in patients undergoing liver resection. B, Immunoblot showing PML, RASSF6, and NLRP12 expression of whole liver lysates isolated of either tumor or tumor‐surrounding liver tissue of five patients undergoing liver transplantation for HCC. The three selected proteins show decreased expression within the tumor tissue when compared to tumor surrounding tissue. C, Graph indicating densitometric analysis of each band, verifying PML, RASSF6, and NLRP12 downregulation within tumor tissue vs tumor‐surrounding liver tissue in 10 patients undergoing liver transplantation for HCC. All figures represent the mean of at least n = 3 samples per experimental group. mRNA is expressed normalized to *GAPDH* (ns, nonsignificant; *P < 0.05, **P < 0.01, ***P < 0.001; error bars indicate SEM). NTT, nontumorous tissue; TST, tumor‐surrounding tissue; TT, tumor tissue

We subsequently analyzed human liver samples for the expression of RASSF6 and NLRP12 in association with the expression of PML. Here, we utilized whole‐liver mRNA from tumor tissue (TT) and tumor‐surrounding tissue (TST) from livers of patients undergoing liver resection for HCC, and compared these findings to liver tissues resected due to various underlying liver diseases, but without the presence of HCC (NTT). We compared mRNA expression of *PML*, *RASSF6,* and *NLRP12* in livers that were resected without evidence of HCC (NTT) to livers that harbored HCC. Of the livers that contained HCC, we selected TST as well as TT and compared expression of the above‐mentioned genes (Figure 4A). For PML there was no significant difference in expression pattern, even though PML in TT liver tissue was slightly decreased. When we analyzed for *RASSF6* and *NLRP12* expression, we could find a highly significant decrease in both *RASSF6* and *NLRP12* within TST, and predominantly within TT.

These findings were confirmed on a posttranscriptional level (Figure 4B,C; Table S3) in livers of patients undergoing liver transplantation for HCC. Explanted livers were analyzed for protein expression in TT vs TST. Protein expression of PML and RASSF6 is decreased in TT vs TST of five livers explanted due to HCC. Here, NLRP12 expression was equal between TST and TT in the five selected livers (Figure 4B). Quantification of immunoblot of a total of 10 livers confirms stable decrease in PML expression within the TT sample as compared to TST sample (Figure 4C). A significant decrease of RASSF6 in TT samples was found, whereas there was no notable difference for NLRP12 expression on a posttranscriptional level (Figure 4C). Table S3 gives a complete list of underlying liver disease of each selected patient. These data suggest that PML and RASSF6 both play a major role in HCC development, whereas NLRP12 is differentially regulated in TT vs TST liver tissue at least on an RNA level.

### 3.5 Knockdown of *PML*, *NLRP12,* and *RASSF6* leads to increased cell proliferation in vitro

**Figure 5:**
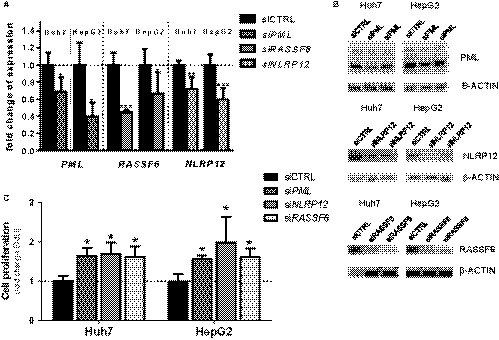
Knockdown of *PML*, *NLRP12,* and *RASSF6* leads to increased cell proliferation in vitro. A, HUH7 and HepG2 cells were either treated with control si‐RNA, or si‐RNA to PML, NLRP12, or RASSF6. mRNA expression confirms successful knockdown of the specific mRNA. B, Immunoblot confirming successful decrease in PML, NLRP12, and RASSF6 on a protein level within HUH7 and HepG2 cells. C, HUH7 and HepG2 cells were treated with si‐RNA to either control, PML, NLRP12, or RASSF6 for 72 h. BrdU incorporation was measured after 2 hours of BrdU treatment. All figures represent the mean of at least n = 3 individual experiments. mRNA is expressed normalized to *GAPDH* (error bars indicate SD)

Since we found a differential expression pattern of PML, RASSF6, and NLRP12, we next aimed to assess the influence of these genes to cell proliferation in vitro. HUH7 and HepG2 cells were transfected using either control si‐RNA, or si‐RNA to *PML*, *NLRP12,* or *RASSF6.* We first confirmed a successful knockdown of these genes as confirmed on RNA and protein level (Figure 5A,B).

To determine whether decreased expression of *PML, NLRP12,* or *RASSF6* leads to increased cell proliferation, cells were transfected using either control si‐RNA, or si‐RNA to *PML*, *NLRP12* or *RASSF6,* after which cells were incubated with BrdU for 2 hours. Cells transfected with siNLRP12 showed a marked increase in cell proliferation when compared to cells transfected with control si‐RNA, or si‐RNA to *PML* or *RASSF6*, as demonstrated via nuclear BrdU incorporation (Figure 5C). The experiment was repeated using alternative siRNAs to *RASSF6* as well as *NLRP12,* reflecting our findings (Figure S3).

These findings indicate a strong correlation between loss of PML, RASSF6, and NLRP12 and increased cell proliferation, both in vitro and in vivo.

## 4 DISCUSSION

Hepatocellular carcinoma is known to be not just one of the most common types of cancer but also one of the most common causes of cancer‐related deaths worldwide. The growing body of evidence for HCV as a major risk factor for the development of liver fibrosis, cirrhosis, and HCC also suggests that the virus plays a direct role in the neoplastic transformation of hepatocytes.^19^ However, the molecular mechanism to this sequence of events is still poorly understood. We have recently conducted a thorough analysis on HCC development under a standardized induction protocol using DEN injections in PML‐deficient, HCV‐transgenic mice.^13^ Our data suggested that PML deficiency increases susceptibility toward carcinogenic stimuli, that HCV promotes carcinogenesis in the liver, and that the oncogenic potential of HCV is supported by an inactivation of PML. Here, our aim was to analyze the spontaneous development of liver tumors in these mice. Interestingly, 40% of the PML^−/−^HCV_tg _mice developed liver tumors by the age of 12 months compared to all other genotypes. Further gene expression profiling of these livers (when compared with the PML^−/−^HCV_tg _livers that did not develop tumors) provided numerous genes associated with increased cell proliferation. RASSF6 has been shown to be expressed in low amounts in HCC, and its overexpression correlates with decreased cell proliferation and invasion in vitro, as well as attenuated tumor growth in a rodent model.^20^ The inhibitory effects are described to be established through suppression of FAK phosphorylation, which in turn leads to decreased MMP2/9 expression.^20^ Our analysis confirms this finding, since we established the downregulation of RASSF6 in liver tissue extracted from liver tumors. Interestingly, not only the tumorous tissue but also the tumor‐surrounding tissue of PML^−/−^HCV_tg _livers, and PML^−/−^HCV_tg _livers without tumor development showed a stepwise decrease in RASSF6 expression. PML‐deficiency in combination with HCV therefore is associated with decreased expression of RASSF6, correlating with increased cell proliferation and tumor growth in vivo and in vitro.

NLRP12, which encodes a negative regulator of innate immunity, has been described to promote specific commensals that can reverse gut inflammation.^21^ However, to the best of our knowledge, its contribution to liver carcinogenesis has not been described so far. Our data suggest that within PML‐deficient and HCV‐transgenic mice, NLRP12 is downregulated in tumor tissue on an RNA level, but not on a posttranscriptional level. This was confirmed in our patient cohort where protein levels were unaltered in contrast to differential expression within tumor tissue vs nontumorous and tumor‐surrounding tissue on an RNA level. Further exploration of this mechanism is intriguing, but beyond the scope of this paper.

Our in vitro data confirm the increase in cell proliferation when either PML, RASSF6, or NLRP12 are downregulated. We chose to leave the cells HCV‐negative in order to assess the unaltered effects of each gene alone.

The correlation of tumor abundance as well as PML deficiency as well as downregulated RASSF6 and NLRP12 expression was confirmed in our patient cohort. As an important point to mention, we chose to investigate liver tissue from patients with various underlying liver diseases, and patients undergoing liver transplantation as well as liver resection. This selection might have blunted the overall effect of gene and protein expression as demonstrated in our analysis. The majority of our patients were HCV positive. However, the heterogeneous underlying diseases reflect the reality of clinical presentation. Future prospective studies will be performed in order to further distinguish between underlying liver disease and correlation with expression patterns of selected genes.

Taken together, this study provides further insight into the role of PML fostering a permissive milieu for the liver toward tumor development, as well as supporting HCV‐related HCC development.

## CONFLICT OF INTEREST

None declared.
