# Supplementary figures and images for: Promyelocytic leukemia protein deficiency leads to spontaneous formation of liver tumors in hepatitis C virus transgenic mice
Source: Cancer Med. 2019 May 29;8(8):3793–802. doi: 10.1002/cam4.2162 (PMC6639172; doi:10.1002/cam4.2162)

**A**

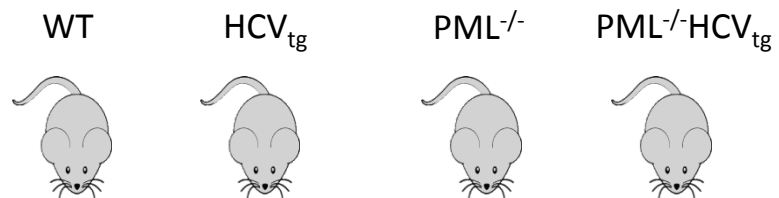

**B**

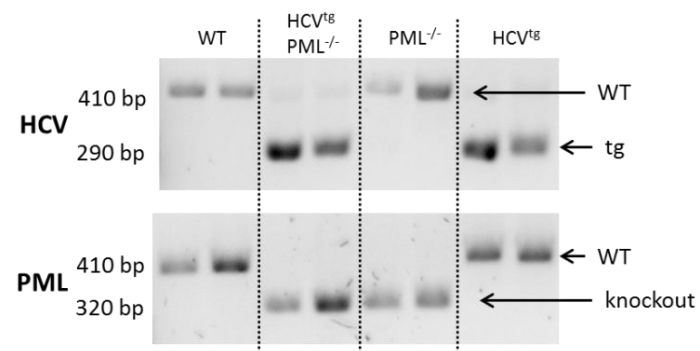

Supplement: Supplementary file 1 [file CAM4-8-3793-s001.pdf]

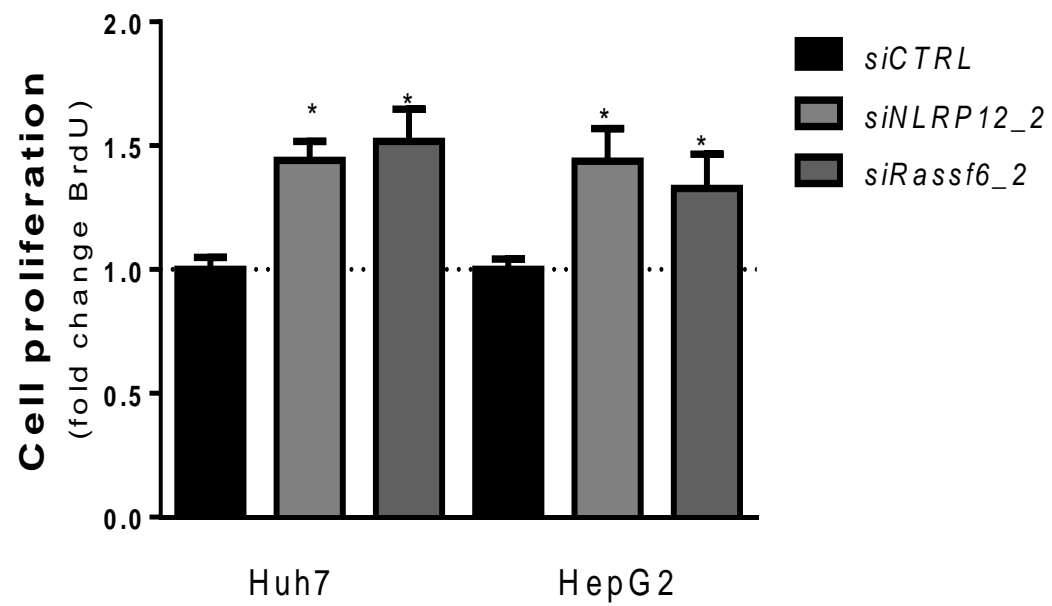

Supplement: Supplementary file 3 [file CAM4-8-3793-s003.pdf]
