# Supplementary material for: Promyelocytic leukemia protein deficiency leads to spontaneous formation of liver tumors in hepatitis C virus transgenic mice
Source: Cancer Med. 2019 May 29;8(8):3793–802. doi: 10.1002/cam4.2162 (PMC6639172; doi:10.1002/cam4.2162)
Supplement: Supplementary file 4 [file CAM4-8-3793-s004.docx]

**Supplementary Table 1. List of Primers**

| **Genotyping** | | |
| --- | --- | --- |
| **Target** | **Name** | **Sequence (5'-3')** |
| HCV | NS4b fwd | TATTGCCTGACAAGAGGCAGTGTGGTTATC |
|  | NS4b rev | GATGAAATTCCACATGTGCTTTGCCCAG |
|  | mFas1 | TCCCTTAAACAGCAGTGCTGTG |
|  | mFas3 | TAGCAAATCTAGGCATTAACAGTG |
| PML | PML-R | TTGGACTTGCGCGTACTGTC |
|  | PML-F1 | TTTCAGTTTCTGCGCTGCC |
|  | neo1500 | ATCGCCTTCTATCGCCTTCTTGACGAG |
| **Quantitative rt-PCR** | | |
| **Species** | **Name** | **Sequence (5'-3')** |
| murine | mACTB fwd | GGCTGTATTCCCCTCCATCG |
|  | mACTB rev | CAGTTGGTAACAATGCCATGT |
|  | mRassf6 fwd | GATCTGTTGAACACACCGGAA |
|  | mRassf6 rev | CGCTGATACGGTAAAGGTCATC |
|  | mNlrp12 fwd | GGATGGCCTCTATCGACTGTC |
|  | mNlrp12 rev | CCTCTGCAATCCCCAGGAATAA |
| human | hGAPDH fwd | CAATGACCCCTTCATTGACC |
|  | hGAPDH rev | GATCTCGCTCCTGGAAGATG |
|  | hRassf6 fwd | ACAGGACCCAGATTCCTATGTC |
|  | hRassf6 rev | GCTGCTTCACTCATGGTTCTAT |
|  | hPML | Qiagen Hs_PML_2_SG  QuantiTect Primer Assay #QT01841945 |
|  |  |  |
|  | hNlrp12 fwd | ACCAGACCTTGACCGACCTT |
|  | hNlrp12 rev | GAGGACTCGGAGTTTGCAGC |

**Supplementary Table 2. Patient Characteristics RNA Analysis**

| **NTT** | **Number of Patients** | **Proportion** |
| --- | --- | --- |
| Colorectal liver metastasis | 6 | 25.0% |
| HCV | 4 | 16.7% |
| Alcoholic steatohepatitis | 1 | 4.2% |
| Cholangiocellular Carcinoma | 3 | 12.5% |
| Focal nodular hyperplasia | 2 | 8.3% |
| Acute liver failure | 2 | 8.3% |
| Klatskin tumor | 2 | 8.3% |
| Adenocarcinoma | 1 | 4.2% |
| Non-alcoholic steatohepatitis | 1 | 4.2% |
| Primary biliary cirrhosis | 2 | 8.3% |
|  |  |  |
| **TST/TT** | **Number of Patients** | **Proportion** |
| HCV | 23 | 74.2% |
| HBV | 3 | 9.7% |
| Non-alcoholic steatohepatitis | 5 | 16.1% |
| Alcoholic steatohepatitis | 2 | 6.5% |
| Cryptogenic liver disease | 6 | 19.4% |

**Supplementary Table 3. Patient Characteristics Protein Analysis**

|  | **Patient number** |  | **Underlying Liver Disease** |
| --- | --- | --- | --- |
| **Blot 1** | p1 | TST | Cryptogenic cirrhosis |
|  |  | TT |  |
|  | p2 | TST | ASH / NASH |
|  |  | TT |  |
|  | p3 | TST | Primary HCC |
|  |  | TT |  |
|  | p4 | TST | Cryptogenic cirrhosis |
|  |  | TT |  |
|  | p5 | TST | NASH |
|  |  | TT |  |
| **Blot 2** | p6 | TST | NASH |
|  |  | TT |  |
|  | p7 | TST | Haemochromatosis |
|  |  | TT |  |
|  | p8 | TST | HBV |
|  |  | TT |  |
|  | p9 | TST | HBV |
|  |  | TT |  |
|  | p10 | TST | ASH + Hemochromatosis |
|  |  | TT |  |

**Supplementary Table 4. Differentially Expressed Genes**

|  | **WT** | | **NTL** | | **TST** | | **TT** | |  |
| --- | --- | --- | --- | --- | --- | --- | --- | --- | --- |
| **Gene Symbol** | **Average** | **SD** | **Average** | **SD** | **Average** | **SD** | **Average** | **SD** |  |
| **Anapc1** | 1.00 | 0.09 | 1.07 | 0.07 | 1.14 | 0.07 | 1.07 | 0.02 |  |
| **Ap3b1** | 1.00 | 0.06 | 1.36 | 0.13 | 1.28 | 0.10 | 1.05 | 0.06 |  |
| **Apoc2** | 1.00 | 0.14 | 1.68 | 0.25 | 1.43 | 0.15 | 1.35 | 0.07 |  |
| **Ar** | 1.00 | 0.08 | 0.73 | 0.07 | 0.37 | 0.17 | 0.49 | 0,13 |  |
| **Cask** | 1.00 | 0.02 | 1.20 | 0.05 | 1.25 | 0.08 | 1.28 | 0.16 |  |
| **Ceacam1** | 1.00 | 0.19 | 1.46 | 0.07 | 1.45 | 0.21 | 1.01 | 0.29 |  |
| **Cep104** | 1.00 | 0.09 | 1.55 | 0.06 | 1.36 | 0.15 | 1.54 | 0.76 |  |
| **Ciapin1** | 1.00 | 0.08 | 1.17 | 0.05 | 1.07 | 0.27 | 1.06 | 0.21 |  |
| **Cox6b2** | 1.00 | 0.21 | 4.31 | 0.85 | 4.79 | 1.38 | 3.41 | 0.82 |  |
| **Ddx19b** | 1.00 | 0.05 | 1.30 | 0.03 | 1.46 | 0.03 | 1.11 | 0.04 |  |
| **Dstyk** | 1.00 | 0.05 | 1.30 | 0.10 | 1.16 | 0.05 | 1.11 | 0.15 |  |
| **Dstyk** | 1.00 | 0.24 | 1.40 | 0.10 | 1.65 | 0.44 | 1.39 | 0.05 |  |
| **Epm2aip1** | 1.00 | 0.12 | 0.68 | 0.02 | 0.72 | 0.13 | 0.73 | 0.07 |  |
| **Fads6** | 1.00 | 0.19 | 0.70 | 0.06 | 0.73 | 0.19 | 0.69 | 0.11 |  |
| **Fkbp14** | 1.00 | 0.10 | 0.68 | 0.12 | 0.60 | 0.16 | 0.62 | 0.12 |  |
| **Fmn1** | 1.00 | 0.12 | 1.74 | 0.17 | 1.63 | 0.24 | 1.41 | 0.71 |  |
| **Foxn3** | 1.00 | 0.21 | 1.15 | 0.13 | 1.19 | 0.06 | 1.19 | 0.15 |  |
| **Gsta2** | 1.00 | 0.05 | 0.59 | 0.02 | 0.37 | 0.11 | 0.43 | 0.08 |  |
| **Hsph1** | 1.00 | 0.08 | 0.52 | 0.06 | 1.60 | 1.60 | 1.89 | 1.43 |  |
| **Igfbp4** | 1.00 | 0.12 | 1.20 | 0.04 | 1.30 | 0.10 | 1.27 | 0.10 |  |
| **Igsf23** | 1.00 | 0.22 | 3.78 | 0.10 | 4.16 | 0.13 | 3.41 | 1.26 |  |
| **Kdm1a** | 1.00 | 0.10 | 1.25 | 0.02 | 0.98 | 0.07 | 1.19 | 0.38 |  |
| **Mapkapk3** | 1.00 | 0.21 | 0.56 | 0.17 | 0.46 | 0.13 | 1.21 | 0.84 |  |
| **Mppe1** | 1.00 | 0.07 | 0.66 | 0.02 | 0.75 | 0.07 | 0.70 | 0.08 |  |
| **Mri1** | 1.00 | 0.16 | 1.47 | 0.20 | 1.64 | 0.10 | 1.37 | 0.01 |  |
| **Ncald** | 1.00 | 0.07 | 0.70 | 0.14 | 0.68 | 0.11 | 0.87 | 0.49 |  |
| **Ndufb10** | 1.00 | 0.05 | 1.31 | 0.06 | 1.18 | 0.04 | 1.24 | 0.42 |  |
| **Nfe2l2** | 1.00 | 0.02 | 0.69 | 0.12 | 0.53 | 0.07 | 0.63 | 0.27 |  |
| **Nlrp12** | 1.00 | 0.14 | 0.03 | 0.01 | 0.04 | 0.01 | 0.03 | 0.00 |  |
| **Ogdh** | 1.00 | 0.05 | 1.49 | 0.09 | 1.41 | 0.13 | 2.14 | 1.19 |  |
| **Parp3** | 1.00 | 0.16 | 0.40 | 0.11 | 0.48 | 0.09 | 0.48 | 0.08 |  |
| **Pde7a** | 1.00 | 0.13 | 0.55 | 0.09 | 0.64 | 0.09 | 1.97 | 1.26 |  |
| **Pik3r4** | 1.00 | 0.04 | 0.64 | 0.05 | 0.70 | 0.09 | 0.65 | 0.10 |  |
| **Ppm1l** | 1.00 | 0.32 | 0.97 | 0.15 | 1.21 | 0.26 | 1.46 | 1.09 |  |
| **Ppp5c** | 1.00 | 0.03 | 1.10 | 0.22 | 1.14 | 0.39 | 1.04 | 0.37 |  |
| **Rassf6** | 1.00 | 0.15 | 0.55 | 0.02 | 0.52 | 0.15 | 0.42 | 0.12 |  |
| **Rassf7** | 1.00 | 0.08 | 0.74 | 0.01 | 0.80 | 0.10 | 0.65 | 0.15 |  |
| **Sfxn1** | 1.00 | 0.11 | 0.80 | 0.06 | 0.87 | 0.19 | 0.77 | 0.16 |  |
| **Slc46a1** | 1.00 | 0.11 | 0.61 | 0.07 | 0.67 | 0.08 | 0.65 | 0.24 |  |
| **Smad3** | 1.00 | 0.16 | 1.43 | 0.18 | 1.40 | 0.27 | 1.39 | 0.39 |  |
| **Sord** | 1.00 | 0.55 | 0.56 | 0.10 | 0.55 | 0.30 | 0.50 | 0.19 |  |
| **Sort1** | 1.00 | 0.21 | 1.57 | 0.16 | 2.26 | 0.15 | 1.59 | 0.42 |  |
| **.Sulf2** | 1.00 | 0.23 | 0.48 | 0.07 | 0.39 | 0.23 | 0.39 | 0.12 |  |
| **Tcn2** | 1.00 | 0.25 | 0.78 | 0.08 | 0.68 | 0.14 | 0.70 | 0.07 |  |
| **Ubiad1** | 1.00 | 0.13 | 0.52 | 0.01 | 0.40 | 0.07 | 0.43 | 0.10 |  |
| **Vamp3** | 1.00 | 0.10 | 2.52 | 0.60 | 1.87 | 0.32 | 2.88 | 1.07 |  |
| **Xdh** | 1.00 | 0.09 | 0.68 | 0.13 | 0.47 | 0.04 | 0.52 | 0.08 |  |
| **Zdhhc21** | 1.00 | 0.10 | 1.37 | 0.13 | 1.43 | 0.10 | 1.65 | 0.30 |  |
|  |  |  |  |  |  |  |  |  |  |
|  |  |  |  |  |  |  |  |  |  |

**Supplementary Table 5. Gene set enrichment analysis**

| **WT vs NTT** | | | | | |
| --- | --- | --- | --- | --- | --- |
| Gene Set database | Enriched in | Gene set | NES | p-value | FDR |
| Biocarta | WT | DC PATHWAY | 1.48 | 0.000 | 1.00 |
|  |  | P53 PATHWAY | 1.40 | 0.000 | 1.00 |
|  |  | ARF PATHWAY | 1.36 | 0.000 | 1.00 |
|  |  | WNT PATHWAY | 1.36 | 0.000 | 1.00 |
|  |  | RACCYCD PATHWAY | 1.35 | 0.000 | 0.94 |
|  |  | TOLL PATHWAY | 1.30 | 0.000 | 0.90 |
|  |  | P38MAPK PATHWAY | 1.29 | 0.000 | 0.91 |
|  |  | CARM ER PATHWAY | 1.37 | 0.066 | 1.00 |
|  |  | GABA PATHWAY | 1.35 | 0.089 | 0.79 |
|  |  | NEUROTRANSMITTERS PATHWAY | 1.24 | 0.100 | 0.98 |
|  |  | P27 PATHWAY | 1.35 | 0.107 | 0.85 |
|  |  | IL1R PATHWAY | 1.41 | 0.110 | 1.00 |
|  |  | G2 PATHWAY | 1.22 | 0.110 | 0.98 |
|  |  | G1 PATHWAY | 1.36 | 0.111 | 1.00 |
|  |  | DNAFRAGMENT PATHWAY | 1.33 | 0.111 | 0.87 |
|  |  | ATM PATHWAY | 1.29 | 0.112 | 0.86 |
|  |  | IL12 PATHWAY | 1.03 | 0.112 | 0.98 |
|  |  | INFLAM PATHWAY | 1.37 | 0.114 | 1.00 |
|  |  | BLYMPHOCYTE PATHWAY | 1.26 | 0.114 | 0.95 |
|  |  | CHREBP2 PATHWAY | 1.11 | 0.183 | 1.00 |
|  |  | NFKB PATHWAY | 1.31 | 0.185 | 0.93 |
|  |  | 41BB PATHWAY | 1.12 | 0.187 | 1.00 |
|  |  | EPONFKB PATHWAY | 1.03 | 0.202 | 0.97 |
|  |  | CD40 PATHWAY | 1.17 | 0.211 | 0.98 |
|  |  | VIP PATHWAY | 1.21 | 0.218 | 0.99 |
|  |  | CARDIACEGF PATHWAY | 1.19 | 0.218 | 0.99 |
|  |  | MAPK PATHWAY | 1.16 | 0.220 | 1.00 |
|  |  | CLASSIC PATHWAY | 1.20 | 0.257 | 0.98 |
|  |  | CFTR PATHWAY | 1.09 | 0.274 | 1.00 |
|  |  | CBL PATHWAY | 1.18 | 0.295 | 0.98 |
|  |  | PTC1 PATHWAY | 1.09 | 0.300 | 1.00 |
|  |  | AHSP PATHWAY | 1.08 | 0.303 | 1.00 |
|  |  | ARAP PATHWAY | 1.07 | 0.305 | 1.00 |
|  |  | PYK2 PATHWAY | 1.05 | 0.306 | 1.00 |
|  |  | CALCINEURIN PATHWAY | 1.02 | 0.324 | 0.97 |
|  |  | CCR5 PATHWAY | 1.10 | 0.326 | 1.00 |
|  |  | P53HYPOXIA PATHWAY | 1.22 | 0.327 | 1.00 |
|  |  | PGC1A PATHWAY | 1.06 | 0.328 | 1.00 |
|  |  | AKAP95 PATHWAY | 0.99 | 0.334 | 0.97 |
|  |  | STEM PATHWAY | 1.06 | 0.371 | 1.00 |
|  |  | IL22BP PATHWAY | 1.02 | 0.380 | 0.96 |
|  |  | NKCELLS PATHWAY | 0.95 | 0.393 | 1.00 |
|  |  | SPRY PATHWAY | 1.03 | 0.414 | 0.98 |
|  |  | CYTOKINE PATHWAY | 1.04 | 0.415 | 1.00 |
|  |  | CELLCYCLE PATHWAY | 1.09 | 0.429 | 1.00 |
|  |  | GRANULOCYTES PATHWAY | 1.04 | 0.444 | 1.00 |
|  |  | HSP27 PATHWAY | 1.01 | 0.448 | 0.97 |
|  |  | LAIR PATHWAY | 0.99 | 0.448 | 0.99 |
|  |  | BAD PATHWAY | 1.05 | 0.452 | 1.00 |
|  |  | HDAC PATHWAY | 0.93 | 0.461 | 1.00 |
|  |  | ACE2 PATHWAY | 0.92 | 0.466 | 1.00 |
|  |  | COMP PATHWAY | 0.87 | 0.482 | 1.00 |
|  |  | FAS PATHWAY | 0.94 | 0.509 | 1.00 |
|  |  | FREE PATHWAY | 1.09 | 0.511 | 1.00 |
|  |  | FMLP PATHWAY | 1.04 | 0.517 | 0.99 |
|  |  | TNFR2 PATHWAY | 0.83 | 0.546 | 1.00 |
|  |  | TID PATHWAY | 0.88 | 0.576 | 1.00 |
|  |  | STRESS PATHWAY | 0.89 | 0.592 | 1.00 |
|  |  | PML PATHWAY | 0.87 | 0.598 | 1.00 |
|  |  | SRCRPTP PATHWAY | 0.87 | 0.607 | 1.00 |
|  |  | SODD PATHWAY | 0.72 | 0.615 | 1.00 |
|  |  | CASPASE PATHWAY | 0.96 | 0.617 | 1.00 |
|  |  | RB PATHWAY | 1.00 | 0.650 | 0.99 |
|  |  | TCYTOTOXIC PATHWAY | 0.74 | 0.666 | 1.00 |
|  |  | MITOCHONDRIA PATHWAY | 0.83 | 0.676 | 1.00 |
|  |  | NO2IL12 PATHWAY | 0.72 | 0.683 | 1.00 |
|  |  | MONOCYTE PATHWAY | 0.73 | 0.687 | 1.00 |
|  |  | AMI PATHWAY | 0.78 | 0.687 | 1.00 |
|  |  | PLCE PATHWAY | 0.85 | 0.692 | 1.00 |
|  |  | D4GDI PATHWAY | 0.81 | 0.697 | 1.00 |
|  |  | EGFR SMRTE PATHWAY | 0.89 | 0.700 | 1.00 |
|  |  | AKAPCENTROSOME PATHWAY | 0.76 | 0.702 | 1.00 |
|  |  | PAR1 PATHWAY | 0.81 | 0.706 | 1.00 |
|  |  | CDMAC PATHWAY | 0.67 | 0.713 | 1.00 |
|  |  | AT1R PATHWAY | 0.90 | 0.713 | 1.00 |
|  |  | TOB1 PATHWAY | 0.79 | 0.717 | 1.00 |
|  |  | RANKL PATHWAY | 0.81 | 0.723 | 1.00 |
|  |  | DEATH PATHWAY | 0.89 | 0.728 | 1.00 |
|  |  | HIVNEF PATHWAY | 0.87 | 0.731 | 1.00 |
|  |  | CTL PATHWAY | 0.84 | 0.756 | 1.00 |
|  |  | TNFR1 PATHWAY | 0.75 | 0.757 | 1.00 |
|  |  | THELPER PATHWAY | 0.55 | 0.784 | 1.00 |
|  |  | LEPTIN PATHWAY | 0.65 | 0.790 | 1.00 |
|  |  | RAC1 PATHWAY | 0.76 | 0.791 | 1.00 |
|  |  | NO1 PATHWAY | 0.79 | 0.802 | 1.00 |
|  |  | MTA3 PATHWAY | 0.73 | 0.803 | 1.00 |
|  |  | EDG1 PATHWAY | 0.63 | 0.803 | 1.00 |
|  |  | NTHI PATHWAY | 0.73 | 0.807 | 1.00 |
|  |  | RHO PATHWAY | 0.77 | 0.807 | 1.00 |
|  |  | ACH PATHWAY | 0.77 | 0.808 | 1.00 |
|  |  | TALL1 PATHWAY | 0.60 | 0.811 | 1.00 |
|  |  | CACAM PATHWAY | 0.63 | 0.812 | 1.00 |
|  |  | CTCF PATHWAY | 0.79 | 0.816 | 1.00 |
|  |  | LYM PATHWAY | 0.68 | 0.835 | 1.00 |
|  |  | LECTIN PATHWAY | 0.63 | 0.889 | 1.00 |
|  |  | MEF2D PATHWAY | 0.52 | 0.889 | 1.00 |
|  |  | BIOPEPTIDES PATHWAY | 0.74 | 0.889 | 1.00 |
|  |  | CTLA4 PATHWAY | 0.44 | 0.891 | 1.00 |
|  |  | RELA PATHWAY | 0.66 | 0.892 | 1.00 |
|  |  | ARENRF2 PATHWAY | 0.47 | 0.896 | 1.00 |
|  |  | ETS PATHWAY | 0.64 | 0.898 | 1.00 |
|  |  | KERATINOCYTE PATHWAY | 0.54 | 0.898 | 1.00 |
|  |  | TEL PATHWAY | 0.60 | 0.898 | 1.00 |
|  |  | SPPA PATHWAY | 0.69 | 0.902 | 1.00 |
|  |  | GPCR PATHWAY | 0.67 | 0.902 | 1.00 |
|  |  | CDC42RAC PATHWAY | 0.50 | 0.902 | 1.00 |
|  |  | BCELLSURVIVAL PATHWAY | 0.55 | 0.903 | 1.00 |
|  |  | MAL PATHWAY | 0.58 | 0.903 | 1.00 |
|  |  | BCR PATHWAY | 0.70 | 0.904 | 1.00 |
|  |  |  |  |  |  |
|  | NCL | KREB PATHWAY | -1.39 | 0.000 | 1.00 |
|  |  | ETC PATHWAY | -1.38 | 0.000 | 1.00 |
|  |  | GH PATHWAY | -1.36 | 0.000 | 1.00 |
|  |  | CELL2CELL PATHWAY | -1.35 | 0.000 | 1.00 |
|  |  | MTOR PATHWAY | -1.30 | 0.000 | 1.00 |
|  |  | NDKDYNAMIN PATHWAY | -1.49 | 0.079 | 1.00 |
|  |  | TRKA PATHWAY | -1.25 | 0.094 | 1.00 |
|  |  | CARM1 PATHWAY | -1.31 | 0.110 | 1.00 |
|  |  | IGF1R PATHWAY | -1.19 | 0.147 | 1.00 |
|  |  | CDK5 PATHWAY | -1.15 | 0.168 | 1.00 |
|  |  | TGFB PATHWAY | -1.20 | 0.169 | 1.00 |
|  |  | VEGF PATHWAY | -1.12 | 0.176 | 1.00 |
|  |  | AKAP13 PATHWAY | -1.10 | 0.181 | 1.00 |
|  |  | PTEN PATHWAY | -1.12 | 0.184 | 1.00 |
|  |  | RARRXR PATHWAY | -1.18 | 0.188 | 1.00 |
|  |  | VITCB PATHWAY | -1.06 | 0.201 | 1.00 |
|  |  | PITX2 PATHWAY | -1.16 | 0.207 | 1.00 |
|  |  | IL5 PATHWAY | -1.15 | 0.212 | 1.00 |
|  |  | CXCR4 PATHWAY | -1.05 | 0.244 | 1.00 |
|  |  | EPHA4 PATHWAY | -1.23 | 0.262 | 1.00 |
|  |  | EPO PATHWAY | -1.16 | 0.262 | 1.00 |
|  |  | INSULIN PATHWAY | -1.13 | 0.270 | 1.00 |
|  |  | IGF1 PATHWAY | -1.12 | 0.270 | 1.00 |
|  |  | NGF PATHWAY | -1.06 | 0.270 | 1.00 |
|  |  | DREAM PATHWAY | -1.05 | 0.276 | 1.00 |
|  |  | TPO PATHWAY | -1.16 | 0.287 | 1.00 |
|  |  | CREB PATHWAY | -1.07 | 0.305 | 1.00 |
|  |  | P35ALZHEIMERS PATHWAY | -1.03 | 0.306 | 1.00 |
|  |  | AGR PATHWAY | -1.03 | 0.310 | 1.00 |
|  |  | SARS PATHWAY | -1.06 | 0.336 | 1.00 |
|  |  | FIBRINOLYSIS PATHWAY | -1.05 | 0.340 | 1.00 |
|  |  | PS1 PATHWAY | -1.00 | 0.349 | 1.00 |
|  |  | BARRESTIN PATHWAY | -1.06 | 0.359 | 1.00 |
|  |  | NKT PATHWAY | -0.91 | 0.409 | 1.00 |
|  |  | HER2 PATHWAY | -1.02 | 0.419 | 1.00 |
|  |  | IL4 PATHWAY | -0.93 | 0.419 | 1.00 |
|  |  | EIF PATHWAY | -0.96 | 0.428 | 1.00 |
|  |  | HIF PATHWAY | -1.07 | 0.430 | 1.00 |
|  |  | RANMS PATHWAY | -0.89 | 0.437 | 1.00 |
|  |  | EIF2 PATHWAY | -1.00 | 0.467 | 1.00 |
|  |  | INTEGRIN PATHWAY | -0.97 | 0.477 | 1.00 |
|  |  | EGF PATHWAY | -0.93 | 0.481 | 1.00 |
|  |  | ASBCELL PATHWAY | -1.01 | 0.482 | 1.00 |
|  |  | IL2 PATHWAY | -0.89 | 0.488 | 1.00 |
|  |  | ALK PATHWAY | -1.06 | 0.498 | 1.00 |
|  |  | PTDINS PATHWAY | -0.92 | 0.510 | 1.00 |
|  |  | GCR PATHWAY | -0.82 | 0.514 | 1.00 |
|  |  | IL10 PATHWAY | -0.75 | 0.516 | 1.00 |
|  |  | TH1TH2 PATHWAY | -0.87 | 0.521 | 1.00 |
|  |  | PPARA PATHWAY | -0.94 | 0.546 | 1.00 |
|  |  | PLATELETAPP PATHWAY | -0.89 | 0.552 | 1.00 |
|  |  | IL6 PATHWAY | -0.80 | 0.554 | 1.00 |
|  |  | PARKIN PATHWAY | -0.78 | 0.555 | 1.00 |
|  |  | MET PATHWAY | -0.79 | 0.557 | 1.00 |
|  |  | CSK PATHWAY | -0.93 | 0.559 | 1.00 |
|  |  | BARR MAPK PATHWAY | -0.92 | 0.560 | 1.00 |
|  |  | TFF PATHWAY | -0.82 | 0.566 | 1.00 |
|  |  | PDGF PATHWAY | -0.79 | 0.577 | 1.00 |
|  |  | NFAT PATHWAY | -0.88 | 0.578 | 1.00 |
|  |  | IL2RB PATHWAY | -0.80 | 0.586 | 1.00 |
|  |  | GLEEVEC PATHWAY | -0.83 | 0.591 | 1.00 |
|  |  | EXTRINSIC PATHWAY | -0.91 | 0.604 | 1.00 |
|  |  | SHH PATHWAY | -0.91 | 0.608 | 1.00 |
|  |  | UCALPAIN PATHWAY | -0.85 | 0.608 | 1.00 |
|  |  | SKP2E2F PATHWAY | -0.64 | 0.621 | 1.00 |
|  |  | EIF4 PATHWAY | -0.96 | 0.627 | 1.00 |
|  |  | TCRA PATHWAY | -0.81 | 0.649 | 1.00 |
|  |  | SET PATHWAY | -0.80 | 0.649 | 1.00 |
|  |  | CK1 PATHWAY | -0.78 | 0.665 | 1.00 |
|  |  | ATRBRCA PATHWAY | -0.81 | 0.672 | 1.00 |
|  |  | ECM PATHWAY | -0.86 | 0.679 | 1.00 |
|  |  | ERK PATHWAY | -0.87 | 0.685 | 1.00 |
|  |  | GATA3 PATHWAY | -0.88 | 0.697 | 1.00 |
|  |  | VDR PATHWAY | -0.79 | 0.703 | 1.00 |
|  |  | HCMV PATHWAY | -0.81 | 0.703 | 1.00 |
|  |  | IL3 PATHWAY | -0.94 | 0.709 | 1.00 |
|  |  | IL17 PATHWAY | -0.79 | 0.712 | 1.00 |
|  |  | IL7 PATHWAY | -0.61 | 0.715 | 1.00 |
|  |  | RAS PATHWAY | -0.79 | 0.717 | 1.00 |
|  |  | PROTEASOME PATHWAY | -0.93 | 0.734 | 1.00 |
|  |  | ACTINY PATHWAY | -0.70 | 0.764 | 1.00 |
|  |  | FEEDER PATHWAY | -0.69 | 0.764 | 1.00 |
|  |  | SALMONELLA PATHWAY | -0.62 | 0.764 | 1.00 |
|  |  | CCR3 PATHWAY | -0.73 | 0.771 | 1.00 |
|  |  | GSK3 PATHWAY | -0.74 | 0.783 | 1.00 |
|  |  | RAB PATHWAY | -0.59 | 0.787 | 1.00 |
|  |  | BARRESTIN SRC PATHWAY | -0.59 | 0.794 | 1.00 |
|  |  | AGPCR PATHWAY | -0.60 | 0.804 | 1.00 |
|  |  | ERYTH PATHWAY | -0.63 | 0.807 | 1.00 |
|  |  | MCALPAIN PATHWAY | -0.73 | 0.812 | 1.00 |
|  |  | MCM PATHWAY | -0.74 | 0.887 | 1.00 |
|  |  | FCER1 PATHWAY | -0.48 | 0.887 | 1.00 |
|  |  | MPR PATHWAY | -0.65 | 0.889 | 1.00 |
|  |  | IGF1MTOR PATHWAY | -0.64 | 0.889 | 1.00 |
|  |  | NOS1 PATHWAY | -0.63 | 0.889 | 1.00 |
|  |  | CHEMICAL PATHWAY | -0.59 | 0.889 | 1.00 |
|  |  | CERAMIDE PATHWAY | -0.54 | 0.889 | 1.00 |
|  |  | GLYCOLYSIS PATHWAY | -0.54 | 0.889 | 1.00 |
|  |  | TCR PATHWAY | -0.47 | 0.889 | 0.99 |
|  |  | STATHMIN PATHWAY | -0.54 | 0.893 | 1.00 |
|  |  | INTRINSIC PATHWAY | -0.78 | 0.894 | 1.00 |
|  |  | RNA PATHWAY | -0.61 | 0.894 | 1.00 |
|  |  | TCAPOPTOSIS PATHWAY | -0.58 | 0.898 | 1.00 |
|  |  | MYOSIN PATHWAY | -0.76 | 0.899 | 1.00 |
|  |  | AKT PATHWAY | -0.71 | 0.900 | 1.00 |
|  |  | ERK5 PATHWAY | -0.47 | 0.901 | 1.00 |
|  |  | LONGEVITY PATHWAY | -0.53 | 0.902 | 1.00 |
|  |  | NUCLEARRS PATHWAY | -0.78 | 0.904 | 1.00 |
|  |  |  |  |  |  |
| KEGG | WT | MATURITY ONSET DIABETES OF THE YOUNG | 1.81 | 0.000 | 0.28 |
|  |  | BLADDER CANCER | 1.57 | 0.000 | 0.89 |
|  |  | RIG I LIKE RECEPTOR SIGNALING PATHWAY | 1.44 | 0.000 | 1.00 |
|  |  | ONE CARBON POOL BY FOLATE | 1.43 | 0.000 | 1.00 |
|  |  | NON HOMOLOGOUS END JOINING | 1.40 | 0.000 | 1.00 |
|  |  | MELANOMA | 1.38 | 0.000 | 1.00 |
|  |  | NICOTINATE AND NICOTINAMIDE METABOLISM | 1.30 | 0.000 | 1.00 |
|  |  | RIBOFLAVIN METABOLISM | 1.21 | 0.000 | 1.00 |
|  |  | TYPE II DIABETES MELLITUS | 1.23 | 0.136 | 1.00 |
|  |  | AUTOIMMUNE THYROID DISEASE | 1.30 | 0.139 | 0.97 |
|  |  | AMYOTROPHIC LATERAL SCLEROSIS ALS | 1.21 | 0.140 | 0.93 |
|  |  | NOD LIKE RECEPTOR SIGNALING PATHWAY | 1.01 | 0.140 | 0.88 |
|  |  | OTHER GLYCAN DEGRADATION | 1.34 | 0.141 | 1.00 |
|  |  | FRUCTOSE AND MANNOSE METABOLISM | 1.32 | 0.142 | 1.00 |
|  |  | MAPK SIGNALING PATHWAY | 1.14 | 0.143 | 1.00 |
|  |  | CYTOKINE CYTOKINE RECEPTOR INTERACTION | 1.29 | 0.143 | 0.90 |
|  |  | OOCYTE MEIOSIS | 1.24 | 0.143 | 1.00 |
|  |  | TOLL LIKE RECEPTOR SIGNALING PATHWAY | 1.18 | 0.143 | 0.95 |
|  |  | BASE EXCISION REPAIR | 1.23 | 0.144 | 1.00 |
|  |  | GAP JUNCTION | 1.15 | 0.144 | 1.00 |
|  |  | LINOLEIC ACID METABOLISM | 1.06 | 0.168 | 0.94 |
|  |  | CALCIUM SIGNALING PATHWAY | 1.10 | 0.188 | 1.00 |
|  |  | NITROGEN METABOLISM | 1.15 | 0.191 | 1.00 |
|  |  | NEUROACTIVE LIGAND RECEPTOR INTERACTION | 1.05 | 0.203 | 0.89 |
|  |  | CIRCADIAN RHYTHM MAMMAL | 1.11 | 0.212 | 1.00 |
|  |  | PROGESTERONE MEDIATED OOCYTE MATURATION | 1.21 | 0.221 | 0.98 |
|  |  | EPITHELIAL CELL SIGNALING IN HELICOBACTER PYLORI INFECTION | 1.10 | 0.226 | 1.00 |
|  |  | CYTOSOLIC DNA SENSING PATHWAY | 1.12 | 0.226 | 1.00 |
|  |  | GLYCOSAMINOGLYCAN DEGRADATION | 1.31 | 0.237 | 1.00 |
|  |  | GLYCOSPHINGOLIPID BIOSYNTHESIS GANGLIO SERIES | 1.08 | 0.264 | 1.00 |
|  |  | LONG TERM DEPRESSION | 1.06 | 0.270 | 0.92 |
|  |  | TASTE TRANSDUCTION | 1.06 | 0.305 | 0.95 |
|  |  | P53 SIGNALING PATHWAY | 1.34 | 0.312 | 1.00 |
|  |  | JAK STAT SIGNALING PATHWAY | 1.20 | 0.314 | 0.92 |
|  |  | LYSOSOME | 1.06 | 0.315 | 0.97 |
|  |  | CELL CYCLE | 1.23 | 0.317 | 1.00 |
|  |  | ACUTE MYELOID LEUKEMIA | 1.00 | 0.329 | 0.86 |
|  |  | NATURAL KILLER CELL MEDIATED CYTOTOXICITY | 0.98 | 0.333 | 0.86 |
|  |  | REGULATION OF ACTIN CYTOSKELETON | 1.01 | 0.335 | 0.88 |
|  |  | MISMATCH REPAIR | 1.09 | 0.337 | 1.00 |
|  |  | GRAFT VERSUS HOST DISEASE | 1.04 | 0.337 | 0.87 |
|  |  | ANTIGEN PROCESSING AND PRESENTATION | 1.08 | 0.339 | 0.97 |
|  |  | GLIOMA | 1.00 | 0.341 | 0.87 |
|  |  | RIBOSOME | 0.96 | 0.342 | 0.81 |
|  |  | SMALL CELL LUNG CANCER | 1.01 | 0.353 | 0.90 |
|  |  | CHEMOKINE SIGNALING PATHWAY | 0.87 | 0.353 | 0.88 |
|  |  | PANCREATIC CANCER | 1.08 | 0.360 | 0.99 |
|  |  | OLFACTORY TRANSDUCTION | 1.05 | 0.375 | 0.88 |
|  |  | SELENOAMINO ACID METABOLISM | 1.07 | 0.380 | 0.97 |
|  |  | THYROID CANCER | 1.04 | 0.413 | 0.88 |
|  |  | CHRONIC MYELOID LEUKEMIA | 0.97 | 0.414 | 0.83 |
|  |  | MTOR SIGNALING PATHWAY | 0.99 | 0.420 | 0.87 |
|  |  | SYSTEMIC LUPUS ERYTHEMATOSUS | 0.88 | 0.424 | 0.87 |
|  |  | DRUG METABOLISM CYTOCHROME P450 | 1.09 | 0.426 | 1.00 |
|  |  | GLYCOSAMINOGLYCAN BIOSYNTHESIS KERATAN SULFATE | 1.02 | 0.433 | 0.92 |
|  |  | FC GAMMA R MEDIATED PHAGOCYTOSIS | 0.90 | 0.435 | 0.87 |
|  |  | LEISHMANIA INFECTION | 1.06 | 0.442 | 0.90 |
|  |  | FOCAL ADHESION | 0.86 | 0.442 | 0.88 |
|  |  | GLYCOSPHINGOLIPID BIOSYNTHESIS GLOBO SERIES | 0.90 | 0.443 | 0.87 |
|  |  | ARRHYTHMOGENIC RIGHT VENTRICULAR CARDIOMYOPATHY ARVC | 0.97 | 0.445 | 0.84 |
|  |  | PROSTATE CANCER | 0.96 | 0.455 | 0.83 |
|  |  | ARACHIDONIC ACID METABOLISM | 0.98 | 0.487 | 0.86 |
|  |  | GNRH SIGNALING PATHWAY | 1.03 | 0.488 | 0.90 |
|  |  | TAURINE AND HYPOTAURINE METABOLISM | 1.00 | 0.490 | 0.87 |
|  |  | HISTIDINE METABOLISM | 0.87 | 0.491 | 0.88 |
|  |  | UBIQUITIN MEDIATED PROTEOLYSIS | 0.94 | 0.501 | 0.85 |
|  |  | GALACTOSE METABOLISM | 0.97 | 0.525 | 0.84 |
|  |  | PURINE METABOLISM | 0.86 | 0.526 | 0.88 |
|  |  | PATHWAYS IN CANCER | 0.97 | 0.527 | 0.85 |
|  |  | VEGF SIGNALING PATHWAY | 0.78 | 0.529 | 0.94 |
|  |  | HOMOLOGOUS RECOMBINATION | 0.96 | 0.534 | 0.84 |
|  |  | APOPTOSIS | 0.90 | 0.539 | 0.86 |
|  |  | VASOPRESSIN REGULATED WATER REABSORPTION | 0.94 | 0.558 | 0.84 |
|  |  | GLYCINE SERINE AND THREONINE METABOLISM | 0.81 | 0.567 | 0.91 |
|  |  | ALLOGRAFT REJECTION | 0.88 | 0.570 | 0.89 |
|  |  | PROXIMAL TUBULE BICARBONATE RECLAMATION | 0.85 | 0.607 | 0.88 |
|  |  | NOTCH SIGNALING PATHWAY | 0.98 | 0.607 | 0.87 |
|  |  | ALDOSTERONE REGULATED SODIUM REABSORPTION | 0.93 | 0.611 | 0.84 |
|  |  | INTESTINAL IMMUNE NETWORK FOR IGA PRODUCTION | 0.93 | 0.614 | 0.84 |
|  |  | COLORECTAL CANCER | 0.83 | 0.615 | 0.89 |
|  |  | SPHINGOLIPID METABOLISM | 0.82 | 0.615 | 0.90 |
|  |  | ARGININE AND PROLINE METABOLISM | 0.97 | 0.619 | 0.85 |
|  |  | O GLYCAN BIOSYNTHESIS | 0.88 | 0.621 | 0.88 |
|  |  | GLYCEROLIPID METABOLISM | 0.96 | 0.624 | 0.81 |
|  |  | METABOLISM OF XENOBIOTICS BY CYTOCHROME P450 | 0.87 | 0.636 | 0.89 |
|  |  | SULFUR METABOLISM | 0.73 | 0.711 | 0.96 |
|  |  | INSULIN SIGNALING PATHWAY | 0.81 | 0.724 | 0.90 |
|  |  | PRIMARY IMMUNODEFICIENCY | 0.71 | 0.726 | 0.94 |
|  |  | PHOSPHATIDYLINOSITOL SIGNALING SYSTEM | 0.68 | 0.728 | 0.92 |
|  |  | ECM RECEPTOR INTERACTION | 0.73 | 0.730 | 0.96 |
|  |  | DNA REPLICATION | 0.72 | 0.735 | 0.96 |
|  |  | VIRAL MYOCARDITIS | 0.67 | 0.737 | 0.93 |
|  |  | HYPERTROPHIC CARDIOMYOPATHY HCM | 0.82 | 0.746 | 0.90 |
|  |  | DRUG METABOLISM OTHER ENZYMES | 0.69 | 0.812 | 0.93 |
|  |  | GLYCOSAMINOGLYCAN BIOSYNTHESIS HEPARAN SULFATE | 0.83 | 0.816 | 0.90 |
|  |  | LEUKOCYTE TRANSENDOTHELIAL MIGRATION | 0.70 | 0.816 | 0.93 |
|  |  | DILATED CARDIOMYOPATHY | 0.76 | 0.817 | 0.96 |
|  |  | GLUTATHIONE METABOLISM | 0.64 | 0.822 | 0.93 |
|  |  | FC EPSILON RI SIGNALING PATHWAY | 0.69 | 0.828 | 0.92 |
|  |  | SPLICEOSOME | 0.72 | 0.828 | 0.95 |
|  |  | NON SMALL CELL LUNG CANCER | 0.74 | 0.832 | 0.97 |
|  |  | STEROID HORMONE BIOSYNTHESIS | 0.75 | 0.833 | 0.96 |
|  |  | AMINO SUGAR AND NUCLEOTIDE SUGAR METABOLISM | 0.71 | 0.834 | 0.93 |
|  |  | ETHER LIPID METABOLISM | 0.70 | 0.838 | 0.93 |
|  |  | LONG TERM POTENTIATION | 0.67 | 0.922 | 0.92 |
|  |  | GLYCOSAMINOGLYCAN BIOSYNTHESIS CHONDROITIN SULFATE | 0.74 | 0.924 | 0.97 |
|  |  | HEMATOPOIETIC CELL LINEAGE | 0.53 | 0.924 | 0.98 |
|  |  |  |  |  |  |
|  | NCL | OXIDATIVE PHOSPHORYLATION | -1.76 | 0.000 | 0.32 |
|  |  | HUNTINGTONS DISEASE | -1.51 | 0.000 | 0.78 |
|  |  | PARKINSONS DISEASE | -1.50 | 0.000 | 0.58 |
|  |  | LYSINE DEGRADATION | -1.42 | 0.000 | 0.83 |
|  |  | VALINE LEUCINE AND ISOLEUCINE BIOSYNTHESIS | -1.42 | 0.000 | 0.67 |
|  |  | PENTOSE AND GLUCURONATE INTERCONVERSIONS | -1.34 | 0.000 | 0.90 |
|  |  | RNA DEGRADATION | -1.25 | 0.000 | 1.00 |
|  |  | ALZHEIMERS DISEASE | -1.24 | 0.000 | 0.99 |
|  |  | CARDIAC MUSCLE CONTRACTION | -1.34 | 0.083 | 1.00 |
|  |  | PPAR SIGNALING PATHWAY | -1.23 | 0.182 | 0.97 |
|  |  | FATTY ACID METABOLISM | -1.30 | 0.185 | 1.00 |
|  |  | VALINE LEUCINE AND ISOLEUCINE DEGRADATION | -1.19 | 0.192 | 1.00 |
|  |  | TRYPTOPHAN METABOLISM | -1.16 | 0.192 | 1.00 |
|  |  | RETINOL METABOLISM | -1.18 | 0.200 | 1.00 |
|  |  | INOSITOL PHOSPHATE METABOLISM | -1.06 | 0.235 | 1.00 |
|  |  | PRIMARY BILE ACID BIOSYNTHESIS | -1.06 | 0.274 | 1.00 |
|  |  | PORPHYRIN AND CHLOROPHYLL METABOLISM | -1.07 | 0.292 | 1.00 |
|  |  | CITRATE CYCLE TCA CYCLE | -1.27 | 0.294 | 1.00 |
|  |  | CELL ADHESION MOLECULES CAMS | -1.03 | 0.299 | 1.00 |
|  |  | PANTOTHENATE AND COA BIOSYNTHESIS | -1.10 | 0.304 | 1.00 |
|  |  | ABC TRANSPORTERS | -1.05 | 0.377 | 1.00 |
|  |  | PEROXISOME | -1.16 | 0.390 | 1.00 |
|  |  | BETA ALANINE METABOLISM | -1.10 | 0.390 | 1.00 |
|  |  | GLYCOSYLPHOSPHATIDYLINOSITOL GPI ANCHOR BIOSYNTHESIS | -1.03 | 0.396 | 1.00 |
|  |  | VIBRIO CHOLERAE INFECTION | -0.97 | 0.404 | 1.00 |
|  |  | PYRUVATE METABOLISM | -1.00 | 0.433 | 1.00 |
|  |  | BUTANOATE METABOLISM | -0.93 | 0.433 | 1.00 |
|  |  | AMINOACYL TRNA BIOSYNTHESIS | -0.98 | 0.495 | 1.00 |
|  |  | BASAL TRANSCRIPTION FACTORS | -0.97 | 0.500 | 1.00 |
|  |  | VASCULAR SMOOTH MUSCLE CONTRACTION | -0.94 | 0.500 | 1.00 |
|  |  | ENDOMETRIAL CANCER | -0.91 | 0.505 | 1.00 |
|  |  | RNA POLYMERASE | -0.90 | 0.511 | 1.00 |
|  |  | AXON GUIDANCE | -0.97 | 0.513 | 1.00 |
|  |  | TGF BETA SIGNALING PATHWAY | -0.89 | 0.515 | 1.00 |
|  |  | PROPANOATE METABOLISM | -1.04 | 0.518 | 1.00 |
|  |  | ASCORBATE AND ALDARATE METABOLISM | -0.94 | 0.526 | 1.00 |
|  |  | TYROSINE METABOLISM | -0.94 | 0.532 | 1.00 |
|  |  | FOLATE BIOSYNTHESIS | -1.00 | 0.534 | 1.00 |
|  |  | CYSTEINE AND METHIONINE METABOLISM | -0.94 | 0.535 | 1.00 |
|  |  | ENDOCYTOSIS | -0.98 | 0.580 | 1.00 |
|  |  | ADIPOCYTOKINE SIGNALING PATHWAY | -0.94 | 0.605 | 1.00 |
|  |  | T CELL RECEPTOR SIGNALING PATHWAY | -0.83 | 0.608 | 1.00 |
|  |  | B CELL RECEPTOR SIGNALING PATHWAY | -0.79 | 0.608 | 1.00 |
|  |  | LIMONENE AND PINENE DEGRADATION | -0.90 | 0.610 | 1.00 |
|  |  | GLYCOSPHINGOLIPID BIOSYNTHESIS LACTO AND NEOLACTO SERIES | -0.89 | 0.620 | 0.97 |
|  |  | BIOSYNTHESIS OF UNSATURATED FATTY ACIDS | -0.93 | 0.623 | 1.00 |
|  |  | DORSO VENTRAL AXIS FORMATION | -0.83 | 0.627 | 1.00 |
|  |  | RENAL CELL CARCINOMA | -0.95 | 0.629 | 1.00 |
|  |  | ERBB SIGNALING PATHWAY | -0.93 | 0.629 | 1.00 |
|  |  | HEDGEHOG SIGNALING PATHWAY | -0.86 | 0.678 | 1.00 |
|  |  | GLYOXYLATE AND DICARBOXYLATE METABOLISM | -0.87 | 0.706 | 1.00 |
|  |  | TERPENOID BACKBONE BIOSYNTHESIS | -0.82 | 0.711 | 1.00 |
|  |  | NEUROTROPHIN SIGNALING PATHWAY | -0.91 | 0.715 | 1.00 |
|  |  | STEROID BIOSYNTHESIS | -0.82 | 0.715 | 1.00 |
|  |  | STARCH AND SUCROSE METABOLISM | -0.74 | 0.726 | 1.00 |
|  |  | ADHERENS JUNCTION | -0.89 | 0.811 | 0.98 |
|  |  | SNARE INTERACTIONS IN VESICULAR TRANSPORT | -0.73 | 0.816 | 1.00 |
|  |  | WNT SIGNALING PATHWAY | -0.90 | 0.816 | 1.00 |
|  |  | PENTOSE PHOSPHATE PATHWAY | -0.57 | 0.816 | 1.00 |
|  |  | PRION DISEASES | -0.58 | 0.818 | 1.00 |
|  |  | COMPLEMENT AND COAGULATION CASCADES | -0.70 | 0.819 | 1.00 |
|  |  | MELANOGENESIS | -0.84 | 0.827 | 1.00 |
|  |  | RENIN ANGIOTENSIN SYSTEM | -0.70 | 0.833 | 1.00 |
|  |  | BASAL CELL CARCINOMA | -0.68 | 0.834 | 1.00 |
|  |  | TIGHT JUNCTION | -0.74 | 0.837 | 1.00 |
|  |  | GLYCEROPHOSPHOLIPID METABOLISM | -0.73 | 0.916 | 1.00 |
|  |  | PYRIMIDINE METABOLISM | -0.56 | 0.921 | 1.00 |
|  |  | PROTEASOME | -0.71 | 0.922 | 1.00 |
|  |  | NUCLEOTIDE EXCISION REPAIR | -0.66 | 0.922 | 1.00 |
|  |  | ALANINE ASPARTATE AND GLUTAMATE METABOLISM | -0.65 | 0.922 | 1.00 |
|  |  | N GLYCAN BIOSYNTHESIS | -0.61 | 0.922 | 1.00 |
|  |  | PATHOGENIC ESCHERICHIA COLI INFECTION | -0.48 | 0.922 | 1.00 |
|  |  | GLYCOLYSIS GLUCONEOGENESIS | -0.48 | 0.922 | 1.00 |
|  |  | PROTEIN EXPORT | -0.34 | 0.922 | 1.00 |
|  |  | ASTHMA | -0.56 | 0.922 | 1.00 |
|  |  | TYPE I DIABETES MELLITUS | -0.55 | 0.922 | 1.00 |
|  |  | REGULATION OF AUTOPHAGY | -0.67 | 0.926 | 1.00 |
|  |  | ALPHA LINOLENIC ACID METABOLISM | -0.74 | 0.926 | 1.00 |
|  |  | PHENYLALANINE METABOLISM | -0.80 | 0.928 | 1.00 |
|  |  |  |  |  |  |
| LIVER CANCER SIGNATURES | WT | KUROKAWA LIVER CANCER EARLY RECURRENCE | 1.47 | 0.000 | 0.37 |
|  |  | CHIANG LIVER CANCER SUBCLASS INTERFERON | 1.10 | 0.181 | 0.70 |
|  |  | LEE LIVER CANCER HEPATOBLAST | 1.35 | 0.191 | 0.59 |
|  |  | CHIANG LIVER CANCER SUBCLASS PROLIFERATION | 0.96 | 0.285 | 0.80 |
|  |  | CK19SIGNATURE FDR010 | 1.15 | 0.292 | 0.99 |
|  |  | CAIRO LIVER CANCER HEPATOBLAST | 0.99 | 0.382 | 0.99 |
|  |  | YAMASHITA LIVER CANCER EPCAM | 0.96 | 0.494 | 0.72 |
|  |  | WOO LIVER CANCER CHOLANGIOCA LIKE UP | 1.12 | 0.495 | 0.81 |
|  |  | COULOUARN LIVER CANCER TGF BETA LATE VS EARLY | 0.97 | 0.504 | 0.90 |
|  |  | WOO LIVER CANCER RECURRENCE | 0.86 | 0.507 | 0.77 |
|  |  | HOSHIDA S1 | 0.83 | 0.516 | 0.69 |
|  |  | ROESSLER LIVER CANCER METASTASIS UP | 0.86 | 0.598 | 0.71 |
|  |  | NOVAK MET | 0.93 | 0.689 | 0.70 |
|  |  | LEE LIVER CANCER POOR SURVIVAL | 0.55 | 0.882 | 0.97 |
|  |  |  |  |  |  |
|  | NCL | YE LIVER CANCER INTRAHEPATIC METS | -1.30 | 0.000 | 1.00 |
|  |  | WANG LIVER CANCER RECURRENCE | -1.15 | 0.181 | 0.74 |
|  |  | BOYAULT LIVER CANCER SUBCLASS G12 | -1.28 | 0.184 | 0.67 |
|  |  | CHIANG LIVER CANCER SUBCLASS POLYSOMY7 | -1.14 | 0.184 | 0.48 |
|  |  | BOYAULT LIVER CANCER SUBCLASS G56 | -1.15 | 0.227 | 0.57 |
|  |  | CHIANG LIVER CANCER SUBCLASS CTNNB1 | -1.04 | 0.370 | 0.66 |
|  |  | VI SIGNATURE FDR0 ABSDIFF100 | -0.92 | 0.475 | 0.62 |
|  |  | HOSHIDA S2 | -1.01 | 0.498 | 0.61 |
|  |  | HOSHIDA S3 | -0.93 | 0.542 | 0.69 |
|  |  | BOYAULT LIVER CANCER SUBCLASS G3 | -0.70 | 0.768 | 0.90 |
|  |  | YOSHIOKA LIVER CANCER EARLY RECURRENCE | -0.63 | 0.789 | 0.92 |
|  |  |  |  |  |  |
| **NTT vs TST** | | | | | |
| Gene Set database | Enriched in | Gene set | NES | p-value | FDR |
| Biocarta | NCL | BARR MAPK PATHWAY | 1.68 | 0.000 | 0.15 |
|  |  | GPCR PATHWAY | 1.60 | 0.000 | 0.39 |
|  |  | ARENRF2 PATHWAY | 1.55 | 0.000 | 0.39 |
|  |  | BARRESTIN PATHWAY | 1.55 | 0.000 | 0.40 |
|  |  | PS1 PATHWAY | 1.52 | 0.000 | 0.40 |
|  |  | AGPCR PATHWAY | 1.51 | 0.000 | 0.39 |
|  |  | BARRESTIN SRC PATHWAY | 1.50 | 0.000 | 0.39 |
|  |  | AGR PATHWAY | 1.47 | 0.000 | 0.46 |
|  |  | CCR3 PATHWAY | 1.46 | 0.000 | 0.45 |
|  |  | CXCR4 PATHWAY | 1.45 | 0.000 | 0.46 |
|  |  | FMLP PATHWAY | 1.45 | 0.000 | 0.42 |
|  |  | GLYCOLYSIS PATHWAY | 1.42 | 0.000 | 0.49 |
|  |  | GH PATHWAY | 1.39 | 0.000 | 0.59 |
|  |  | STATHMIN PATHWAY | 1.35 | 0.000 | 0.60 |
|  |  | DREAM PATHWAY | 1.34 | 0.000 | 0.59 |
|  |  | CSK PATHWAY | 1.33 | 0.000 | 0.57 |
|  |  | PTDINS PATHWAY | 1.31 | 0.000 | 0.58 |
|  |  | TCR PATHWAY | 1.31 | 0.000 | 0.55 |
|  |  | ERK PATHWAY | 1.28 | 0.000 | 0.58 |
|  |  | IL2 PATHWAY | 1.27 | 0.000 | 0.61 |
|  |  | MPR PATHWAY | 1.21 | 0.000 | 0.66 |
|  |  | PLATELETAPP PATHWAY | 1.33 | 0.089 | 0.58 |
|  |  | CREB PATHWAY | 1.23 | 0.095 | 0.67 |
|  |  | PITX2 PATHWAY | 1.37 | 0.096 | 0.63 |
|  |  | NFAT PATHWAY | 1.17 | 0.103 | 0.72 |
|  |  | ECM PATHWAY | 1.34 | 0.104 | 0.60 |
|  |  | TCRA PATHWAY | 1.16 | 0.106 | 0.72 |
|  |  | EIF2 PATHWAY | 1.52 | 0.106 | 0.45 |
|  |  | IGF1R PATHWAY | 1.32 | 0.108 | 0.60 |
|  |  | ETC PATHWAY | 1.31 | 0.109 | 0.56 |
|  |  | CARM1 PATHWAY | 1.23 | 0.110 | 0.64 |
|  |  | AKAP13 PATHWAY | 1.30 | 0.110 | 0.55 |
|  |  | ERK5 PATHWAY | 1.19 | 0.112 | 0.69 |
|  |  | ACE2 PATHWAY | 1.30 | 0.112 | 0.53 |
|  |  | PPARA PATHWAY | 1.37 | 0.113 | 0.60 |
|  |  | ALK PATHWAY | 1.36 | 0.195 | 0.60 |
|  |  | RB PATHWAY | 1.22 | 0.200 | 0.64 |
|  |  | NGF PATHWAY | 1.12 | 0.200 | 0.79 |
|  |  | AKAP95 PATHWAY | 1.12 | 0.214 | 0.77 |
|  |  | IL17 PATHWAY | 1.05 | 0.216 | 0.91 |
|  |  | KREB PATHWAY | 1.23 | 0.216 | 0.68 |
|  |  | EIF4 PATHWAY | 1.19 | 0.218 | 0.68 |
|  |  | IGF1 PATHWAY | 1.20 | 0.219 | 0.68 |
|  |  | MAL PATHWAY | 1.15 | 0.219 | 0.73 |
|  |  | VIP PATHWAY | 1.15 | 0.225 | 0.73 |
|  |  | ASBCELL PATHWAY | 1.24 | 0.226 | 0.68 |
|  |  | VITCB PATHWAY | 1.31 | 0.227 | 0.60 |
|  |  | AT1R PATHWAY | 1.06 | 0.230 | 0.91 |
|  |  | BCR PATHWAY | 1.16 | 0.230 | 0.73 |
|  |  | TPO PATHWAY | 1.05 | 0.235 | 0.92 |
|  |  | GATA3 PATHWAY | 1.25 | 0.272 | 0.67 |
|  |  | MTOR PATHWAY | 1.05 | 0.283 | 0.89 |
|  |  | TOB1 PATHWAY | 1.05 | 0.302 | 0.88 |
|  |  | NKCELLS PATHWAY | 1.18 | 0.304 | 0.73 |
|  |  | INSULIN PATHWAY | 1.23 | 0.306 | 0.65 |
|  |  | AKAPCENTROSOME PATHWAY | 1.07 | 0.320 | 0.89 |
|  |  | ATRBRCA PATHWAY | 1.22 | 0.320 | 0.64 |
|  |  | PROTEASOME PATHWAY | 1.05 | 0.322 | 0.90 |
|  |  | NUCLEARRS PATHWAY | 1.16 | 0.335 | 0.73 |
|  |  | PARKIN PATHWAY | 1.23 | 0.381 | 0.65 |
|  |  | ARF PATHWAY | 0.95 | 0.388 | 0.92 |
|  |  | CARDIACEGF PATHWAY | 1.12 | 0.389 | 0.77 |
|  |  | CELL2CELL PATHWAY | 0.97 | 0.394 | 0.96 |
|  |  | ETS PATHWAY | 0.98 | 0.401 | 0.97 |
|  |  | CHEMICAL PATHWAY | 1.02 | 0.407 | 0.88 |
|  |  | SODD PATHWAY | 0.96 | 0.422 | 0.95 |
|  |  | TCAPOPTOSIS PATHWAY | 1.04 | 0.422 | 0.88 |
|  |  | NO2IL12 PATHWAY | 0.96 | 0.422 | 0.93 |
|  |  | BAD PATHWAY | 0.86 | 0.442 | 0.98 |
|  |  | CK1 PATHWAY | 1.09 | 0.443 | 0.87 |
|  |  | PYK2 PATHWAY | 1.00 | 0.455 | 0.94 |
|  |  | EPHA4 PATHWAY | 0.90 | 0.501 | 0.98 |
|  |  | GRANULOCYTES PATHWAY | 0.98 | 0.502 | 0.96 |
|  |  | VEGF PATHWAY | 1.03 | 0.503 | 0.89 |
|  |  | IL12 PATHWAY | 0.90 | 0.513 | 0.96 |
|  |  | THELPER PATHWAY | 0.97 | 0.515 | 0.95 |
|  |  | TCYTOTOXIC PATHWAY | 0.95 | 0.515 | 0.91 |
|  |  | IL4 PATHWAY | 0.86 | 0.527 | 0.99 |
|  |  | DC PATHWAY | 0.92 | 0.533 | 0.98 |
|  |  | D4GDI PATHWAY | 0.86 | 0.534 | 0.98 |
|  |  | LEPTIN PATHWAY | 0.95 | 0.545 | 0.91 |
|  |  | EIF PATHWAY | 0.88 | 0.588 | 0.98 |
|  |  | FIBRINOLYSIS PATHWAY | 0.92 | 0.589 | 0.97 |
|  |  | SARS PATHWAY | 0.96 | 0.600 | 0.93 |
|  |  | PGC1A PATHWAY | 0.99 | 0.602 | 0.95 |
|  |  | FCER1 PATHWAY | 0.91 | 0.607 | 0.98 |
|  |  | DEATH PATHWAY | 0.79 | 0.610 | 0.99 |
|  |  | MCM PATHWAY | 0.88 | 0.611 | 0.98 |
|  |  | CTL PATHWAY | 0.85 | 0.621 | 0.96 |
|  |  | CTLA4 PATHWAY | 1.00 | 0.627 | 0.94 |
|  |  | PTEN PATHWAY | 0.96 | 0.629 | 0.93 |
|  |  | MET PATHWAY | 0.90 | 0.630 | 0.96 |
|  |  | MAPK PATHWAY | 0.87 | 0.644 | 0.99 |
|  |  | MONOCYTE PATHWAY | 0.84 | 0.647 | 0.97 |
|  |  | KERATINOCYTE PATHWAY | 0.80 | 0.657 | 0.99 |
|  |  | SET PATHWAY | 0.83 | 0.692 | 0.96 |
|  |  | CALCINEURIN PATHWAY | 0.84 | 0.701 | 0.97 |
|  |  | G2 PATHWAY | 0.76 | 0.703 | 1.00 |
|  |  | CDMAC PATHWAY | 0.86 | 0.711 | 0.97 |
|  |  | NDKDYNAMIN PATHWAY | 0.76 | 0.714 | 1.00 |
|  |  | CASPASE PATHWAY | 0.71 | 0.717 | 0.96 |
|  |  | PML PATHWAY | 0.83 | 0.721 | 0.96 |
|  |  | IGF1MTOR PATHWAY | 0.70 | 0.721 | 0.96 |
|  |  | MCALPAIN PATHWAY | 0.92 | 0.723 | 0.98 |
|  |  | CERAMIDE PATHWAY | 0.72 | 0.726 | 0.99 |
|  |  | EPO PATHWAY | 0.74 | 0.728 | 1.00 |
|  |  | CDK5 PATHWAY | 0.90 | 0.731 | 0.96 |
|  |  | CYTOKINE PATHWAY | 0.72 | 0.736 | 1.00 |
|  |  | ACH PATHWAY | 0.77 | 0.739 | 1.00 |
|  |  | IL7 PATHWAY | 0.65 | 0.742 | 0.96 |
|  |  | TH1TH2 PATHWAY | 0.73 | 0.752 | 1.00 |
|  |  | GSK3 PATHWAY | 0.85 | 0.766 | 0.97 |
|  |  | HDAC PATHWAY | 0.83 | 0.807 | 0.96 |
|  |  | TFF PATHWAY | 0.71 | 0.809 | 0.97 |
|  |  | ACTINY PATHWAY | 0.67 | 0.813 | 0.98 |
|  |  | SALMONELLA PATHWAY | 0.56 | 0.813 | 0.98 |
|  |  | CCR5 PATHWAY | 0.71 | 0.815 | 0.98 |
|  |  | EDG1 PATHWAY | 0.86 | 0.817 | 0.98 |
|  |  | NKT PATHWAY | 0.71 | 0.818 | 0.97 |
|  |  | INTEGRIN PATHWAY | 0.78 | 0.819 | 1.00 |
|  |  | MITOCHONDRIA PATHWAY | 0.66 | 0.820 | 0.97 |
|  |  | DNAFRAGMENT PATHWAY | 0.62 | 0.820 | 0.98 |
|  |  | LYM PATHWAY | 0.54 | 0.820 | 0.98 |
|  |  | TRKA PATHWAY | 0.79 | 0.920 | 0.99 |
|  |  | LONGEVITY PATHWAY | 0.75 | 0.921 | 1.00 |
|  |  | GCR PATHWAY | 0.71 | 0.921 | 0.98 |
|  |  | MYOSIN PATHWAY | 0.60 | 0.923 | 0.97 |
|  |  | RAB PATHWAY | 0.61 | 0.925 | 0.98 |
|  |  | CDC42RAC PATHWAY | 0.49 | 0.925 | 0.99 |
|  |  | RELA PATHWAY | 0.57 | 0.925 | 0.98 |
|  |  | NOS1 PATHWAY | 0.81 | 0.925 | 0.98 |
|  |  | CTCF PATHWAY | 0.73 | 0.926 | 1.00 |
|  |  | TNFR1 PATHWAY | 0.66 | 0.927 | 0.97 |
|  |  | NTHI PATHWAY | 0.73 | 0.928 | 0.99 |
|  |  | HIVNEF PATHWAY | 0.67 | 0.928 | 0.97 |
|  |  | FAS PATHWAY | 0.72 | 0.929 | 1.00 |
|  |  |  |  |  |  |
|  | TST | MTA3 PATHWAY | -1.57 | 0.000 | 0.57 |
|  |  | GABA PATHWAY | -1.53 | 0.000 | 0.64 |
|  |  | LECTIN PATHWAY | -1.47 | 0.000 | 0.75 |
|  |  | TGFB PATHWAY | -1.41 | 0.000 | 0.71 |
|  |  | CD40 PATHWAY | -1.33 | 0.000 | 0.94 |
|  |  | SHH PATHWAY | -1.31 | 0.000 | 0.93 |
|  |  | CFTR PATHWAY | -1.19 | 0.065 | 0.96 |
|  |  | AKT PATHWAY | -1.26 | 0.089 | 0.82 |
|  |  | EPONFKB PATHWAY | -1.44 | 0.089 | 0.84 |
|  |  | EXTRINSIC PATHWAY | -1.12 | 0.093 | 0.90 |
|  |  | IL5 PATHWAY | -1.26 | 0.106 | 0.87 |
|  |  | IL1R PATHWAY | -1.39 | 0.108 | 0.69 |
|  |  | IL22BP PATHWAY | -1.22 | 0.108 | 0.96 |
|  |  | P27 PATHWAY | -1.29 | 0.110 | 0.82 |
|  |  | G1 PATHWAY | -1.18 | 0.113 | 0.92 |
|  |  | EGFR SMRTE PATHWAY | -1.42 | 0.119 | 0.80 |
|  |  | IL10 PATHWAY | -1.29 | 0.200 | 0.89 |
|  |  | RNA PATHWAY | -1.06 | 0.201 | 0.83 |
|  |  | SKP2E2F PATHWAY | -1.14 | 0.202 | 0.93 |
|  |  | ARAP PATHWAY | -1.18 | 0.206 | 0.90 |
|  |  | STEM PATHWAY | -1.21 | 0.208 | 0.92 |
|  |  | INFLAM PATHWAY | -1.20 | 0.214 | 0.92 |
|  |  | WNT PATHWAY | -1.11 | 0.216 | 0.86 |
|  |  | CHREBP2 PATHWAY | -1.11 | 0.218 | 0.83 |
|  |  | CACAM PATHWAY | -1.30 | 0.218 | 0.89 |
|  |  | RACCYCD PATHWAY | -1.19 | 0.220 | 0.94 |
|  |  | CARM ER PATHWAY | -1.26 | 0.227 | 0.87 |
|  |  | VDR PATHWAY | -1.02 | 0.258 | 0.88 |
|  |  | TID PATHWAY | -1.12 | 0.313 | 0.93 |
|  |  | ERYTH PATHWAY | -1.15 | 0.314 | 0.94 |
|  |  | HCMV PATHWAY | -0.99 | 0.315 | 0.89 |
|  |  | IL3 PATHWAY | -1.15 | 0.319 | 0.95 |
|  |  | NFKB PATHWAY | -1.11 | 0.327 | 0.89 |
|  |  | CLASSIC PATHWAY | -1.11 | 0.393 | 0.91 |
|  |  | PLCE PATHWAY | -1.01 | 0.393 | 0.85 |
|  |  | GLEEVEC PATHWAY | -1.07 | 0.400 | 0.84 |
|  |  | RAS PATHWAY | -0.88 | 0.408 | 0.93 |
|  |  | NEUROTRANSMITTERS PATHWAY | -1.10 | 0.409 | 0.81 |
|  |  | 41BB PATHWAY | -0.97 | 0.412 | 0.90 |
|  |  | IL2RB PATHWAY | -1.03 | 0.413 | 0.87 |
|  |  | PTC1 PATHWAY | -0.95 | 0.414 | 0.91 |
|  |  | HER2 PATHWAY | -1.08 | 0.414 | 0.85 |
|  |  | IL6 PATHWAY | -1.10 | 0.419 | 0.80 |
|  |  | P38MAPK PATHWAY | -1.06 | 0.428 | 0.83 |
|  |  | FREE PATHWAY | -0.96 | 0.477 | 0.89 |
|  |  | RARRXR PATHWAY | -1.01 | 0.490 | 0.86 |
|  |  | LAIR PATHWAY | -0.97 | 0.501 | 0.91 |
|  |  | RHO PATHWAY | -0.86 | 0.502 | 0.97 |
|  |  | COMP PATHWAY | -1.11 | 0.523 | 0.83 |
|  |  | NO1 PATHWAY | -0.89 | 0.525 | 0.95 |
|  |  | ATM PATHWAY | -0.81 | 0.540 | 0.96 |
|  |  | UCALPAIN PATHWAY | -0.93 | 0.546 | 0.94 |
|  |  | TEL PATHWAY | -0.95 | 0.548 | 0.92 |
|  |  | TNFR2 PATHWAY | -0.90 | 0.614 | 0.98 |
|  |  | EGF PATHWAY | -0.90 | 0.621 | 0.96 |
|  |  | INTRINSIC PATHWAY | -0.84 | 0.636 | 0.97 |
|  |  | TOLL PATHWAY | -0.81 | 0.693 | 0.95 |
|  |  | PDGF PATHWAY | -0.80 | 0.714 | 0.95 |
|  |  | HSP27 PATHWAY | -0.66 | 0.714 | 0.98 |
|  |  | HIF PATHWAY | -0.63 | 0.716 | 0.98 |
|  |  | AHSP PATHWAY | -0.74 | 0.721 | 0.98 |
|  |  | SPPA PATHWAY | -0.90 | 0.722 | 0.93 |
|  |  | P35ALZHEIMERS PATHWAY | -0.90 | 0.725 | 0.95 |
|  |  | BIOPEPTIDES PATHWAY | -0.82 | 0.726 | 0.99 |
|  |  | STRESS PATHWAY | -0.84 | 0.740 | 0.96 |
|  |  | P53HYPOXIA PATHWAY | -0.86 | 0.764 | 0.96 |
|  |  | MEF2D PATHWAY | -0.82 | 0.810 | 0.98 |
|  |  | BLYMPHOCYTE PATHWAY | -0.60 | 0.810 | 0.98 |
|  |  | SRCRPTP PATHWAY | -0.53 | 0.812 | 1.00 |
|  |  | PAR1 PATHWAY | -0.78 | 0.815 | 0.96 |
|  |  | TALL1 PATHWAY | -0.71 | 0.816 | 0.96 |
|  |  | RAC1 PATHWAY | -0.73 | 0.818 | 0.96 |
|  |  | RANMS PATHWAY | -0.70 | 0.823 | 0.96 |
|  |  | RANKL PATHWAY | -0.80 | 0.829 | 0.95 |
|  |  | CELLCYCLE PATHWAY | -0.51 | 0.829 | 1.00 |
|  |  | AMI PATHWAY | -0.76 | 0.846 | 0.96 |
|  |  | SPRY PATHWAY | -0.72 | 0.852 | 0.97 |
|  |  | P53 PATHWAY | -0.77 | 0.924 | 0.96 |
|  |  | CBL PATHWAY | -0.62 | 0.924 | 0.98 |
|  |  | FEEDER PATHWAY | -0.49 | 0.924 | 0.99 |
|  |  | BCELLSURVIVAL PATHWAY | -0.73 | 0.925 | 0.97 |
|  |  |  |  |  |  |
| KEGG | NCL | DRUG METABOLISM CYTOCHROME P450 | 1.86 | 0.000 | 0.04 |
|  |  | METABOLISM OF XENOBIOTICS BY CYTOCHROME P450 | 1.77 | 0.000 | 0.04 |
|  |  | PARKINSONS DISEASE | 1.69 | 0.000 | 0.04 |
|  |  | GLYOXYLATE AND DICARBOXYLATE METABOLISM | 1.62 | 0.000 | 0.11 |
|  |  | GLUTATHIONE METABOLISM | 1.56 | 0.000 | 0.19 |
|  |  | ASCORBATE AND ALDARATE METABOLISM | 1.56 | 0.000 | 0.18 |
|  |  | ALANINE ASPARTATE AND GLUTAMATE METABOLISM | 1.47 | 0.000 | 0.46 |
|  |  | PENTOSE AND GLUCURONATE INTERCONVERSIONS | 1.42 | 0.000 | 0.46 |
|  |  | PPAR SIGNALING PATHWAY | 1.42 | 0.000 | 0.44 |
|  |  | PANTOTHENATE AND COA BIOSYNTHESIS | 1.40 | 0.000 | 0.47 |
|  |  | PRIMARY BILE ACID BIOSYNTHESIS | 1.35 | 0.000 | 0.46 |
|  |  | GLYCOSAMINOGLYCAN BIOSYNTHESIS HEPARAN SULFATE | 1.31 | 0.000 | 0.51 |
|  |  | ARGININE AND PROLINE METABOLISM | 1.28 | 0.000 | 0.48 |
|  |  | AMYOTROPHIC LATERAL SCLEROSIS ALS | 1.24 | 0.000 | 0.52 |
|  |  | PYRUVATE METABOLISM | 1.25 | 0.090 | 0.52 |
|  |  | STEROID HORMONE BIOSYNTHESIS | 1.25 | 0.091 | 0.51 |
|  |  | WNT SIGNALING PATHWAY | 1.10 | 0.091 | 0.72 |
|  |  | LYSINE DEGRADATION | 1.27 | 0.092 | 0.49 |
|  |  | VALINE LEUCINE AND ISOLEUCINE DEGRADATION | 1.48 | 0.092 | 0.44 |
|  |  | BUTANOATE METABOLISM | 1.44 | 0.092 | 0.52 |
|  |  | PEROXISOME | 1.44 | 0.092 | 0.48 |
|  |  | CITRATE CYCLE TCA CYCLE | 1.39 | 0.092 | 0.47 |
|  |  | BIOSYNTHESIS OF UNSATURATED FATTY ACIDS | 1.37 | 0.092 | 0.45 |
|  |  | HUNTINGTONS DISEASE | 1.29 | 0.094 | 0.49 |
|  |  | TRYPTOPHAN METABOLISM | 1.62 | 0.095 | 0.09 |
|  |  | FOLATE BIOSYNTHESIS | 1.23 | 0.096 | 0.52 |
|  |  | TYROSINE METABOLISM | 1.26 | 0.097 | 0.50 |
|  |  | GLYCOLYSIS GLUCONEOGENESIS | 1.16 | 0.102 | 0.63 |
|  |  | PROTEASOME | 1.19 | 0.108 | 0.62 |
|  |  | MELANOGENESIS | 1.31 | 0.114 | 0.50 |
|  |  | GLYCEROLIPID METABOLISM | 1.16 | 0.121 | 0.62 |
|  |  | VALINE LEUCINE AND ISOLEUCINE BIOSYNTHESIS | 1.30 | 0.198 | 0.50 |
|  |  | PROPANOATE METABOLISM | 1.27 | 0.198 | 0.47 |
|  |  | BETA ALANINE METABOLISM | 1.18 | 0.203 | 0.61 |
|  |  | FATTY ACID METABOLISM | 1.35 | 0.204 | 0.44 |
|  |  | RETINOL METABOLISM | 1.20 | 0.206 | 0.63 |
|  |  | ALZHEIMERS DISEASE | 1.19 | 0.206 | 0.63 |
|  |  | OXIDATIVE PHOSPHORYLATION | 1.42 | 0.210 | 0.49 |
|  |  | LIMONENE AND PINENE DEGRADATION | 1.30 | 0.210 | 0.51 |
|  |  | STEROID BIOSYNTHESIS | 1.38 | 0.220 | 0.45 |
|  |  | ECM RECEPTOR INTERACTION | 1.12 | 0.222 | 0.69 |
|  |  | RENIN ANGIOTENSIN SYSTEM | 1.17 | 0.228 | 0.61 |
|  |  | PHENYLALANINE METABOLISM | 1.04 | 0.285 | 0.84 |
|  |  | SELENOAMINO ACID METABOLISM | 1.04 | 0.288 | 0.85 |
|  |  | LINOLEIC ACID METABOLISM | 1.29 | 0.299 | 0.47 |
|  |  | DRUG METABOLISM OTHER ENZYMES | 1.16 | 0.301 | 0.61 |
|  |  | BASAL CELL CARCINOMA | 1.16 | 0.308 | 0.65 |
|  |  | FOCAL ADHESION | 1.07 | 0.328 | 0.80 |
|  |  | GLYCEROPHOSPHOLIPID METABOLISM | 1.04 | 0.376 | 0.83 |
|  |  | ABC TRANSPORTERS | 1.04 | 0.391 | 0.81 |
|  |  | HISTIDINE METABOLISM | 1.02 | 0.400 | 0.83 |
|  |  | STARCH AND SUCROSE METABOLISM | 0.97 | 0.418 | 0.88 |
|  |  | NITROGEN METABOLISM | 1.02 | 0.420 | 0.82 |
|  |  | GLYCINE SERINE AND THREONINE METABOLISM | 0.98 | 0.425 | 0.88 |
|  |  | PENTOSE PHOSPHATE PATHWAY | 0.99 | 0.434 | 0.86 |
|  |  | B CELL RECEPTOR SIGNALING PATHWAY | 0.86 | 0.436 | 0.96 |
|  |  | NICOTINATE AND NICOTINAMIDE METABOLISM | 0.98 | 0.438 | 0.89 |
|  |  | VEGF SIGNALING PATHWAY | 0.92 | 0.442 | 0.93 |
|  |  | DORSO VENTRAL AXIS FORMATION | 0.89 | 0.446 | 0.92 |
|  |  | ADIPOCYTOKINE SIGNALING PATHWAY | 1.03 | 0.464 | 0.82 |
|  |  | ARACHIDONIC ACID METABOLISM | 0.97 | 0.503 | 0.88 |
|  |  | TIGHT JUNCTION | 0.96 | 0.525 | 0.88 |
|  |  | SPHINGOLIPID METABOLISM | 1.05 | 0.532 | 0.85 |
|  |  | GALACTOSE METABOLISM | 0.92 | 0.532 | 0.92 |
|  |  | AMINO SUGAR AND NUCLEOTIDE SUGAR METABOLISM | 0.90 | 0.536 | 0.94 |
|  |  | T CELL RECEPTOR SIGNALING PATHWAY | 1.00 | 0.541 | 0.84 |
|  |  | ALLOGRAFT REJECTION | 0.94 | 0.550 | 0.91 |
|  |  | REGULATION OF AUTOPHAGY | 0.86 | 0.610 | 0.95 |
|  |  | PROXIMAL TUBULE BICARBONATE RECLAMATION | 0.90 | 0.611 | 0.95 |
|  |  | MTOR SIGNALING PATHWAY | 0.88 | 0.614 | 0.92 |
|  |  | NEUROACTIVE LIGAND RECEPTOR INTERACTION | 0.85 | 0.615 | 0.95 |
|  |  | GLYCOSAMINOGLYCAN DEGRADATION | 0.79 | 0.617 | 0.94 |
|  |  | HEDGEHOG SIGNALING PATHWAY | 0.84 | 0.620 | 0.95 |
|  |  | TERPENOID BACKBONE BIOSYNTHESIS | 0.80 | 0.627 | 0.97 |
|  |  | ETHER LIPID METABOLISM | 0.83 | 0.631 | 0.92 |
|  |  | AMINOACYL TRNA BIOSYNTHESIS | 0.76 | 0.702 | 0.95 |
|  |  | CYSTEINE AND METHIONINE METABOLISM | 0.95 | 0.710 | 0.88 |
|  |  | CARDIAC MUSCLE CONTRACTION | 0.89 | 0.719 | 0.94 |
|  |  | DNA REPLICATION | 0.85 | 0.721 | 0.96 |
|  |  | CELL ADHESION MOLECULES CAMS | 0.79 | 0.721 | 0.97 |
|  |  | INOSITOL PHOSPHATE METABOLISM | 0.81 | 0.726 | 0.94 |
|  |  | GLIOMA | 0.78 | 0.737 | 0.94 |
|  |  | LEUKOCYTE TRANSENDOTHELIAL MIGRATION | 0.74 | 0.740 | 0.95 |
|  |  | FC GAMMA R MEDIATED PHAGOCYTOSIS | 0.70 | 0.740 | 0.95 |
|  |  | NATURAL KILLER CELL MEDIATED CYTOTOXICITY | 0.70 | 0.740 | 0.94 |
|  |  | PATHWAYS IN CANCER | 0.84 | 0.743 | 0.94 |
|  |  | HOMOLOGOUS RECOMBINATION | 0.75 | 0.743 | 0.95 |
|  |  | FC EPSILON RI SIGNALING PATHWAY | 0.79 | 0.747 | 0.95 |
|  |  | NUCLEOTIDE EXCISION REPAIR | 0.84 | 0.753 | 0.94 |
|  |  | PRIMARY IMMUNODEFICIENCY | 0.63 | 0.762 | 0.94 |
|  |  | PURINE METABOLISM | 0.78 | 0.829 | 0.93 |
|  |  | ARRHYTHMOGENIC RIGHT VENTRICULAR CARDIOMYOPATHY ARVC | 0.69 | 0.832 | 0.95 |
|  |  | NON SMALL CELL LUNG CANCER | 0.76 | 0.841 | 0.96 |
|  |  | RENAL CELL CARCINOMA | 0.89 | 0.845 | 0.94 |
|  |  | VIRAL MYOCARDITIS | 0.65 | 0.853 | 0.94 |
|  |  | DILATED CARDIOMYOPATHY | 0.69 | 0.926 | 0.94 |
|  |  | LONG TERM POTENTIATION | 0.84 | 0.927 | 0.93 |
|  |  | ALPHA LINOLENIC ACID METABOLISM | 0.67 | 0.928 | 0.93 |
|  |  | PYRIMIDINE METABOLISM | 0.64 | 0.928 | 0.94 |
|  |  | VASOPRESSIN REGULATED WATER REABSORPTION | 0.72 | 0.929 | 0.95 |
|  |  | INSULIN SIGNALING PATHWAY | 0.74 | 0.930 | 0.94 |
|  |  | VASCULAR SMOOTH MUSCLE CONTRACTION | 0.79 | 0.931 | 0.96 |
|  |  | PROGESTERONE MEDIATED OOCYTE MATURATION | 0.72 | 0.932 | 0.95 |
|  |  | SMALL CELL LUNG CANCER | 0.68 | 0.933 | 0.94 |
|  |  |  |  |  |  |
|  | TST | GLYCOSPHINGOLIPID BIOSYNTHESIS GANGLIO SERIES | -1.51 | 0.000 | 1.00 |
|  |  | NON HOMOLOGOUS END JOINING | -1.38 | 0.000 | 1.00 |
|  |  | SPLICEOSOME | -1.31 | 0.093 | 1.00 |
|  |  | CHRONIC MYELOID LEUKEMIA | -1.24 | 0.097 | 1.00 |
|  |  | ACUTE MYELOID LEUKEMIA | -1.07 | 0.097 | 1.00 |
|  |  | CIRCADIAN RHYTHM MAMMAL | -1.39 | 0.111 | 1.00 |
|  |  | O GLYCAN BIOSYNTHESIS | -1.29 | 0.116 | 1.00 |
|  |  | MAPK SIGNALING PATHWAY | -1.09 | 0.188 | 1.00 |
|  |  | MELANOMA | -1.08 | 0.190 | 1.00 |
|  |  | RIG I LIKE RECEPTOR SIGNALING PATHWAY | -1.14 | 0.194 | 1.00 |
|  |  | NOD LIKE RECEPTOR SIGNALING PATHWAY | -1.02 | 0.194 | 1.00 |
|  |  | ALDOSTERONE REGULATED SODIUM REABSORPTION | -1.10 | 0.197 | 1.00 |
|  |  | AUTOIMMUNE THYROID DISEASE | -1.14 | 0.200 | 1.00 |
|  |  | ONE CARBON POOL BY FOLATE | -1.13 | 0.201 | 1.00 |
|  |  | RNA DEGRADATION | -1.19 | 0.205 | 1.00 |
|  |  | ASTHMA | -1.26 | 0.209 | 1.00 |
|  |  | MATURITY ONSET DIABETES OF THE YOUNG | -1.15 | 0.213 | 1.00 |
|  |  | GLYCOSAMINOGLYCAN BIOSYNTHESIS KERATAN SULFATE | -1.20 | 0.214 | 1.00 |
|  |  | BASE EXCISION REPAIR | -1.18 | 0.239 | 1.00 |
|  |  | CELL CYCLE | -1.10 | 0.274 | 1.00 |
|  |  | CALCIUM SIGNALING PATHWAY | -1.07 | 0.277 | 1.00 |
|  |  | INTESTINAL IMMUNE NETWORK FOR IGA PRODUCTION | -1.04 | 0.291 | 1.00 |
|  |  | GLYCOSPHINGOLIPID BIOSYNTHESIS GLOBO SERIES | -1.13 | 0.293 | 1.00 |
|  |  | PRION DISEASES | -1.09 | 0.296 | 1.00 |
|  |  | APOPTOSIS | -1.02 | 0.297 | 1.00 |
|  |  | SNARE INTERACTIONS IN VESICULAR TRANSPORT | -1.10 | 0.302 | 1.00 |
|  |  | GNRH SIGNALING PATHWAY | -0.97 | 0.303 | 1.00 |
|  |  | PROSTATE CANCER | -0.96 | 0.370 | 1.00 |
|  |  | SYSTEMIC LUPUS ERYTHEMATOSUS | -1.14 | 0.380 | 1.00 |
|  |  | ENDOCYTOSIS | -0.99 | 0.386 | 1.00 |
|  |  | BLADDER CANCER | -1.00 | 0.386 | 1.00 |
|  |  | ANTIGEN PROCESSING AND PRESENTATION | -0.97 | 0.386 | 1.00 |
|  |  | VIBRIO CHOLERAE INFECTION | -0.99 | 0.393 | 1.00 |
|  |  | CYTOSOLIC DNA SENSING PATHWAY | -1.01 | 0.394 | 1.00 |
|  |  | GLYCOSYLPHOSPHATIDYLINOSITOL GPI ANCHOR BIOSYNTHESIS | -1.04 | 0.404 | 1.00 |
|  |  | AXON GUIDANCE | -1.03 | 0.406 | 1.00 |
|  |  | GRAFT VERSUS HOST DISEASE | -1.08 | 0.415 | 1.00 |
|  |  | P53 SIGNALING PATHWAY | -0.90 | 0.467 | 1.00 |
|  |  | NOTCH SIGNALING PATHWAY | -0.85 | 0.481 | 1.00 |
|  |  | GLYCOSPHINGOLIPID BIOSYNTHESIS LACTO AND NEOLACTO SERIES | -1.01 | 0.486 | 1.00 |
|  |  | N GLYCAN BIOSYNTHESIS | -0.86 | 0.518 | 1.00 |
|  |  | RIBOFLAVIN METABOLISM | -1.04 | 0.527 | 1.00 |
|  |  | EPITHELIAL CELL SIGNALING IN HELICOBACTER PYLORI INFECTION | -0.97 | 0.594 | 1.00 |
|  |  | MISMATCH REPAIR | -0.82 | 0.599 | 1.00 |
|  |  | JAK STAT SIGNALING PATHWAY | -0.95 | 0.600 | 1.00 |
|  |  | LEISHMANIA INFECTION | -0.88 | 0.600 | 1.00 |
|  |  | OLFACTORY TRANSDUCTION | -0.78 | 0.600 | 1.00 |
|  |  | CYTOKINE CYTOKINE RECEPTOR INTERACTION | -0.88 | 0.605 | 1.00 |
|  |  | NEUROTROPHIN SIGNALING PATHWAY | -0.87 | 0.607 | 1.00 |
|  |  | BASAL TRANSCRIPTION FACTORS | -0.78 | 0.616 | 1.00 |
|  |  | PORPHYRIN AND CHLOROPHYLL METABOLISM | -0.94 | 0.619 | 1.00 |
|  |  | GLYCOSAMINOGLYCAN BIOSYNTHESIS CHONDROITIN SULFATE | -0.89 | 0.621 | 1.00 |
|  |  | GAP JUNCTION | -0.90 | 0.704 | 1.00 |
|  |  | PATHOGENIC ESCHERICHIA COLI INFECTION | -0.81 | 0.709 | 1.00 |
|  |  | THYROID CANCER | -0.75 | 0.709 | 0.97 |
|  |  | COMPLEMENT AND COAGULATION CASCADES | -0.88 | 0.719 | 1.00 |
|  |  | SULFUR METABOLISM | -0.76 | 0.721 | 0.98 |
|  |  | PANCREATIC CANCER | -0.77 | 0.723 | 0.99 |
|  |  | TAURINE AND HYPOTAURINE METABOLISM | -0.90 | 0.746 | 1.00 |
|  |  | REGULATION OF ACTIN CYTOSKELETON | -0.83 | 0.811 | 1.00 |
|  |  | HEMATOPOIETIC CELL LINEAGE | -0.79 | 0.811 | 1.00 |
|  |  | CHEMOKINE SIGNALING PATHWAY | -0.67 | 0.811 | 0.97 |
|  |  | ADHERENS JUNCTION | -0.66 | 0.811 | 0.96 |
|  |  | PROTEIN EXPORT | -0.59 | 0.814 | 0.98 |
|  |  | TOLL LIKE RECEPTOR SIGNALING PATHWAY | -0.81 | 0.815 | 1.00 |
|  |  | RNA POLYMERASE | -0.70 | 0.815 | 0.97 |
|  |  | OTHER GLYCAN DEGRADATION | -0.59 | 0.818 | 0.97 |
|  |  | LONG TERM DEPRESSION | -0.79 | 0.818 | 1.00 |
|  |  | TASTE TRANSDUCTION | -0.77 | 0.824 | 0.98 |
|  |  | ENDOMETRIAL CANCER | -0.83 | 0.830 | 1.00 |
|  |  | UBIQUITIN MEDIATED PROTEOLYSIS | -0.87 | 0.833 | 1.00 |
|  |  | PHOSPHATIDYLINOSITOL SIGNALING SYSTEM | -0.77 | 0.834 | 1.00 |
|  |  | LYSOSOME | -0.74 | 0.835 | 0.96 |
|  |  | ERBB SIGNALING PATHWAY | -0.74 | 0.920 | 0.97 |
|  |  | OOCYTE MEIOSIS | -0.80 | 0.921 | 1.00 |
|  |  | RIBOSOME | -0.35 | 0.922 | 1.00 |
|  |  | COLORECTAL CANCER | -0.66 | 0.923 | 0.97 |
|  |  | TYPE II DIABETES MELLITUS | -0.76 | 0.924 | 0.99 |
|  |  | TYPE I DIABETES MELLITUS | -0.68 | 0.925 | 0.98 |
|  |  | FRUCTOSE AND MANNOSE METABOLISM | -0.65 | 0.926 | 0.95 |
|  |  | TGF BETA SIGNALING PATHWAY | -0.72 | 0.927 | 0.97 |
|  |  | HYPERTROPHIC CARDIOMYOPATHY HCM | -0.78 | 0.928 | 1.00 |
|  |  |  |  |  |  |
| LIVER CANCER SIGNATURES | NCL | CHIANG LIVER CANCER SUBCLASS CTNNB1 | 1.62 | 0.000 | 0.07 |
|  |  | BOYAULT LIVER CANCER SUBCLASS G56 | 1.48 | 0.000 | 0.20 |
|  |  | HOSHIDA S3 | 1.43 | 0.000 | 0.20 |
|  |  | YE LIVER CANCER INTRAHEPATIC METS | 1.29 | 0.093 | 0.29 |
|  |  | CHIANG LIVER CANCER SUBCLASS POLYSOMY7 | 1.01 | 0.387 | 0.78 |
|  |  | HOSHIDA S2 | 0.96 | 0.520 | 0.77 |
|  |  | YOSHIOKA LIVER CANCER EARLY RECURRENCE | 0.84 | 0.599 | 0.91 |
|  |  | VI SIGNATURE FDR0 ABSDIFF100 | 0.88 | 0.674 | 0.90 |
|  |  | CHIANG LIVER CANCER SUBCLASS PROLIFERATION | 0.80 | 0.680 | 0.90 |
|  |  | BOYAULT LIVER CANCER SUBCLASS G3 | 0.66 | 0.808 | 1.00 |
|  |  | LEE LIVER CANCER HEPATOBLAST | 0.63 | 0.906 | 0.96 |
|  |  |  |  |  |  |
|  | TST | WANG LIVER CANCER RECURRENCE | -1.23 | 0.089 | 0.99 |
|  |  | KUROKAWA LIVER CANCER EARLY RECURRENCE | -1.51 | 0.100 | 0.35 |
|  |  | NOVAK MET | -1.02 | 0.315 | 1.00 |
|  |  | CK19SIGNATURE FDR010 | -1.06 | 0.396 | 1.00 |
|  |  | BOYAULT LIVER CANCER SUBCLASS G12 | -1.00 | 0.425 | 1.00 |
|  |  | WOO LIVER CANCER CHOLANGIOCA LIKE UP | -0.99 | 0.460 | 0.87 |
|  |  | WOO LIVER CANCER RECURRENCE | -0.87 | 0.460 | 1.00 |
|  |  | HOSHIDA S1 | -0.75 | 0.565 | 0.94 |
|  |  | COULOUARN LIVER CANCER TGF BETA LATE VS EARLY | -0.98 | 0.620 | 0.83 |
|  |  | YAMASHITA LIVER CANCER EPCAM | -0.81 | 0.722 | 1.00 |
|  |  | ROESSLER LIVER CANCER METASTASIS UP | -0.81 | 0.808 | 1.00 |
|  |  | CAIRO LIVER CANCER HEPATOBLAST | -0.79 | 0.911 | 0.97 |
|  |  | CHIANG LIVER CANCER SUBCLASS INTERFERON | -0.63 | 0.911 | 1.00 |
|  |  | LEE LIVER CANCER POOR SURVIVAL | -0.44 | 0.912 | 1.00 |
|  |  |  |  |  |  |
| **TST vs TT** | | | | | |
| Gene Set database | Enriched in | Gene set | NES | p-value | FDR |
| Biocarta | TST | NEUROTRANSMITTERS PATHWAY | 1.57 | 0.000 | 0.49 |
|  |  | STEM PATHWAY | 1.45 | 0.000 | 0.89 |
|  |  | IL10 PATHWAY | 1.38 | 0.087 | 0.98 |
|  |  | CYTOKINE PATHWAY | 1.36 | 0.194 | 0.86 |
|  |  | IL22BP PATHWAY | 1.14 | 0.267 | 1.00 |
|  |  | HER2 PATHWAY | 1.11 | 0.277 | 1.00 |
|  |  | DC PATHWAY | 0.97 | 0.297 | 1.00 |
|  |  | SKP2E2F PATHWAY | 1.09 | 0.307 | 1.00 |
|  |  | EIF2 PATHWAY | 1.15 | 0.341 | 1.00 |
|  |  | SODD PATHWAY | 1.02 | 0.353 | 1.00 |
|  |  | ASBCELL PATHWAY | 1.06 | 0.380 | 1.00 |
|  |  | ERYTH PATHWAY | 0.99 | 0.381 | 1.00 |
|  |  | NUCLEARRS PATHWAY | 1.06 | 0.393 | 1.00 |
|  |  | IL5 PATHWAY | 1.07 | 0.413 | 1.00 |
|  |  | MTA3 PATHWAY | 0.99 | 0.420 | 1.00 |
|  |  | AHSP PATHWAY | 0.99 | 0.422 | 1.00 |
|  |  | LECTIN PATHWAY | 0.95 | 0.471 | 1.00 |
|  |  | INFLAM PATHWAY | 0.94 | 0.495 | 1.00 |
|  |  | NO2IL12 PATHWAY | 0.84 | 0.514 | 1.00 |
|  |  | CLASSIC PATHWAY | 0.72 | 0.582 | 1.00 |
|  |  | EXTRINSIC PATHWAY | 0.77 | 0.607 | 1.00 |
|  |  | AKT PATHWAY | 0.91 | 0.609 | 1.00 |
|  |  | P27 PATHWAY | 0.86 | 0.614 | 1.00 |
|  |  | IL17 PATHWAY | 0.90 | 0.618 | 1.00 |
|  |  | TCAPOPTOSIS PATHWAY | 0.85 | 0.630 | 1.00 |
|  |  | EIF PATHWAY | 0.70 | 0.706 | 1.00 |
|  |  | VDR PATHWAY | 0.72 | 0.711 | 1.00 |
|  |  | COMP PATHWAY | 0.63 | 0.711 | 1.00 |
|  |  | MTOR PATHWAY | 0.59 | 0.796 | 1.00 |
|  |  | TCRA PATHWAY | 0.67 | 0.800 | 1.00 |
|  |  | PML PATHWAY | 0.57 | 0.821 | 1.00 |
|  |  | RARRXR PATHWAY | 0.80 | 0.823 | 1.00 |
|  |  | CDK5 PATHWAY | 0.58 | 0.828 | 1.00 |
|  |  | CTL PATHWAY | 0.53 | 0.828 | 1.00 |
|  |  | LAIR PATHWAY | 0.55 | 0.831 | 1.00 |
|  |  | SET PATHWAY | 0.69 | 0.911 | 1.00 |
|  |  | NFKB PATHWAY | 0.56 | 0.912 | 1.00 |
|  |  | RELA PATHWAY | 0.55 | 0.912 | 1.00 |
|  |  | PTEN PATHWAY | 0.48 | 0.913 | 0.99 |
|  |  | EPONFKB PATHWAY | 0.60 | 0.914 | 1.00 |
|  |  |  |  |  |  |
|  | TT | AKAP95 PATHWAY | -1.56 | 0.000 | 1.00 |
|  |  | SHH PATHWAY | -1.47 | 0.000 | 1.00 |
|  |  | MAL PATHWAY | -1.47 | 0.000 | 1.00 |
|  |  | GPCR PATHWAY | -1.46 | 0.000 | 1.00 |
|  |  | AGPCR PATHWAY | -1.43 | 0.000 | 1.00 |
|  |  | TALL1 PATHWAY | -1.43 | 0.000 | 1.00 |
|  |  | CARM1 PATHWAY | -1.40 | 0.000 | 1.00 |
|  |  | NO1 PATHWAY | -1.40 | 0.000 | 1.00 |
|  |  | CCR3 PATHWAY | -1.39 | 0.000 | 1.00 |
|  |  | NDKDYNAMIN PATHWAY | -1.38 | 0.000 | 1.00 |
|  |  | DREAM PATHWAY | -1.37 | 0.000 | 1.00 |
|  |  | PPARA PATHWAY | -1.36 | 0.000 | 1.00 |
|  |  | ETS PATHWAY | -1.33 | 0.000 | 0.84 |
|  |  | P53HYPOXIA PATHWAY | -1.33 | 0.000 | 0.83 |
|  |  | CHREBP2 PATHWAY | -1.32 | 0.000 | 0.78 |
|  |  | PLCE PATHWAY | -1.32 | 0.000 | 0.77 |
|  |  | EDG1 PATHWAY | -1.30 | 0.000 | 0.76 |
|  |  | G2 PATHWAY | -1.27 | 0.000 | 0.73 |
|  |  | PLATELETAPP PATHWAY | -1.11 | 0.000 | 0.65 |
|  |  | NFAT PATHWAY | -1.35 | 0.082 | 0.91 |
|  |  | AKAPCENTROSOME PATHWAY | -1.20 | 0.083 | 0.65 |
|  |  | HDAC PATHWAY | -1.42 | 0.084 | 1.00 |
|  |  | CREB PATHWAY | -1.36 | 0.084 | 0.96 |
|  |  | CK1 PATHWAY | -1.31 | 0.084 | 0.76 |
|  |  | LONGEVITY PATHWAY | -1.23 | 0.085 | 0.65 |
|  |  | NOS1 PATHWAY | -1.50 | 0.085 | 1.00 |
|  |  | TGFB PATHWAY | -1.28 | 0.085 | 0.75 |
|  |  | ACH PATHWAY | -1.28 | 0.085 | 0.74 |
|  |  | VIP PATHWAY | -1.13 | 0.085 | 0.63 |
|  |  | TOB1 PATHWAY | -1.34 | 0.086 | 0.85 |
|  |  | TEL PATHWAY | -1.28 | 0.087 | 0.76 |
|  |  | ARENRF2 PATHWAY | -1.08 | 0.088 | 0.63 |
|  |  | GLYCOLYSIS PATHWAY | -1.26 | 0.101 | 0.70 |
|  |  | BARR MAPK PATHWAY | -1.31 | 0.102 | 0.76 |
|  |  | BAD PATHWAY | -1.17 | 0.104 | 0.64 |
|  |  | CXCR4 PATHWAY | -1.24 | 0.106 | 0.66 |
|  |  | PARKIN PATHWAY | -1.27 | 0.106 | 0.72 |
|  |  | MPR PATHWAY | -1.17 | 0.106 | 0.64 |
|  |  | ARAP PATHWAY | -1.13 | 0.106 | 0.64 |
|  |  | MAPK PATHWAY | -1.29 | 0.111 | 0.77 |
|  |  | MYOSIN PATHWAY | -1.14 | 0.111 | 0.65 |
|  |  | CFTR PATHWAY | -1.18 | 0.112 | 0.64 |
|  |  | RAC1 PATHWAY | -1.33 | 0.112 | 0.80 |
|  |  | PTDINS PATHWAY | -1.26 | 0.113 | 0.69 |
|  |  | PAR1 PATHWAY | -1.22 | 0.114 | 0.64 |
|  |  | P38MAPK PATHWAY | -1.24 | 0.163 | 0.65 |
|  |  | CTCF PATHWAY | -1.34 | 0.164 | 0.81 |
|  |  | HIF PATHWAY | -1.26 | 0.166 | 0.68 |
|  |  | INSULIN PATHWAY | -1.21 | 0.166 | 0.66 |
|  |  | ECM PATHWAY | -1.31 | 0.167 | 0.74 |
|  |  | ALK PATHWAY | -1.37 | 0.170 | 1.00 |
|  |  | AKAP13 PATHWAY | -1.13 | 0.172 | 0.63 |
|  |  | BCR PATHWAY | -1.20 | 0.173 | 0.67 |
|  |  | CARDIACEGF PATHWAY | -1.20 | 0.173 | 0.66 |
|  |  | CALCINEURIN PATHWAY | -1.18 | 0.173 | 0.63 |
|  |  | AGR PATHWAY | -1.16 | 0.173 | 0.66 |
|  |  | IGF1 PATHWAY | -1.20 | 0.183 | 0.65 |
|  |  | TNFR2 PATHWAY | -1.19 | 0.185 | 0.65 |
|  |  | IGF1R PATHWAY | -1.35 | 0.189 | 0.88 |
|  |  | ERK5 PATHWAY | -1.13 | 0.190 | 0.63 |
|  |  | CCR5 PATHWAY | -1.06 | 0.190 | 0.65 |
|  |  | NTHI PATHWAY | -1.14 | 0.192 | 0.65 |
|  |  | STRESS PATHWAY | -1.02 | 0.192 | 0.70 |
|  |  | PGC1A PATHWAY | -1.10 | 0.192 | 0.65 |
|  |  | INTEGRIN PATHWAY | -1.26 | 0.193 | 0.72 |
|  |  | BARRESTIN SRC PATHWAY | -1.10 | 0.194 | 0.65 |
|  |  | ACE2 PATHWAY | -1.25 | 0.216 | 0.67 |
|  |  | BIOPEPTIDES PATHWAY | -1.12 | 0.234 | 0.62 |
|  |  | MCALPAIN PATHWAY | -1.20 | 0.272 | 0.67 |
|  |  | UCALPAIN PATHWAY | -1.16 | 0.272 | 0.65 |
|  |  | PYK2 PATHWAY | -1.04 | 0.273 | 0.68 |
|  |  | VEGF PATHWAY | -1.19 | 0.274 | 0.66 |
|  |  | HSP27 PATHWAY | -1.08 | 0.277 | 0.64 |
|  |  | MET PATHWAY | -1.16 | 0.281 | 0.65 |
|  |  | MEF2D PATHWAY | -1.24 | 0.284 | 0.67 |
|  |  | STATHMIN PATHWAY | -1.17 | 0.284 | 0.65 |
|  |  | RHO PATHWAY | -1.11 | 0.284 | 0.64 |
|  |  | HCMV PATHWAY | -1.06 | 0.285 | 0.66 |
|  |  | AT1R PATHWAY | -1.23 | 0.285 | 0.65 |
|  |  | CELL2CELL PATHWAY | -1.16 | 0.285 | 0.64 |
|  |  | FIBRINOLYSIS PATHWAY | -1.09 | 0.285 | 0.64 |
|  |  | FAS PATHWAY | -1.09 | 0.285 | 0.64 |
|  |  | FMLP PATHWAY | -1.06 | 0.292 | 0.66 |
|  |  | GCR PATHWAY | -1.25 | 0.295 | 0.66 |
|  |  | CELLCYCLE PATHWAY | -1.02 | 0.298 | 0.70 |
|  |  | VITCB PATHWAY | -1.15 | 0.299 | 0.64 |
|  |  | ERK PATHWAY | -1.13 | 0.301 | 0.64 |
|  |  | CBL PATHWAY | -1.10 | 0.303 | 0.65 |
|  |  | ETC PATHWAY | -1.23 | 0.315 | 0.65 |
|  |  | G1 PATHWAY | -1.09 | 0.325 | 0.64 |
|  |  | 41BB PATHWAY | -1.00 | 0.381 | 0.72 |
|  |  | FCER1 PATHWAY | -1.05 | 0.386 | 0.67 |
|  |  | EPHA4 PATHWAY | -1.18 | 0.390 | 0.63 |
|  |  | ARF PATHWAY | -1.11 | 0.392 | 0.64 |
|  |  | KREB PATHWAY | -1.03 | 0.393 | 0.67 |
|  |  | NGF PATHWAY | -0.85 | 0.399 | 0.73 |
|  |  | TCR PATHWAY | -0.99 | 0.400 | 0.71 |
|  |  | CSK PATHWAY | -1.08 | 0.406 | 0.64 |
|  |  | CD40 PATHWAY | -0.98 | 0.406 | 0.72 |
|  |  | CERAMIDE PATHWAY | -1.11 | 0.416 | 0.64 |
|  |  | PTC1 PATHWAY | -0.82 | 0.417 | 0.76 |
|  |  | CACAM PATHWAY | -1.04 | 0.439 | 0.68 |
|  |  | RAB PATHWAY | -1.14 | 0.441 | 0.64 |
|  |  | ATRBRCA PATHWAY | -0.84 | 0.476 | 0.74 |
|  |  | IL6 PATHWAY | -0.94 | 0.478 | 0.73 |
|  |  | SALMONELLA PATHWAY | -1.05 | 0.479 | 0.66 |
|  |  | IL3 PATHWAY | -0.89 | 0.483 | 0.72 |
|  |  | NKCELLS PATHWAY | -0.98 | 0.484 | 0.71 |
|  |  | GATA3 PATHWAY | -1.00 | 0.486 | 0.72 |
|  |  | TCYTOTOXIC PATHWAY | -0.94 | 0.488 | 0.73 |
|  |  | THELPER PATHWAY | -0.92 | 0.488 | 0.73 |
|  |  | IL12 PATHWAY | -0.95 | 0.492 | 0.73 |
|  |  | RB PATHWAY | -0.95 | 0.494 | 0.73 |
|  |  | TH1TH2 PATHWAY | -0.83 | 0.496 | 0.77 |
|  |  | PS1 PATHWAY | -1.04 | 0.497 | 0.68 |
|  |  | SPRY PATHWAY | -1.03 | 0.498 | 0.68 |
|  |  | SPPA PATHWAY | -0.98 | 0.502 | 0.71 |
|  |  | P53 PATHWAY | -0.84 | 0.502 | 0.75 |
|  |  | P35ALZHEIMERS PATHWAY | -0.82 | 0.504 | 0.77 |
|  |  | GLEEVEC PATHWAY | -0.94 | 0.506 | 0.73 |
|  |  | ATM PATHWAY | -1.00 | 0.508 | 0.71 |
|  |  | FEEDER PATHWAY | -0.99 | 0.514 | 0.70 |
|  |  | RANMS PATHWAY | -0.93 | 0.516 | 0.73 |
|  |  | BLYMPHOCYTE PATHWAY | -0.95 | 0.521 | 0.73 |
|  |  | CHEMICAL PATHWAY | -0.99 | 0.529 | 0.71 |
|  |  | IL2 PATHWAY | -0.97 | 0.529 | 0.70 |
|  |  | PITX2 PATHWAY | -0.93 | 0.579 | 0.73 |
|  |  | LYM PATHWAY | -0.89 | 0.580 | 0.73 |
|  |  | RAS PATHWAY | -0.89 | 0.581 | 0.72 |
|  |  | TID PATHWAY | -0.95 | 0.582 | 0.73 |
|  |  | PDGF PATHWAY | -0.88 | 0.584 | 0.72 |
|  |  | TPO PATHWAY | -0.76 | 0.584 | 0.82 |
|  |  | EPO PATHWAY | -0.88 | 0.595 | 0.72 |
|  |  | MCM PATHWAY | -0.90 | 0.596 | 0.74 |
|  |  | NKT PATHWAY | -0.98 | 0.598 | 0.71 |
|  |  | CDC42RAC PATHWAY | -0.92 | 0.598 | 0.73 |
|  |  | MONOCYTE PATHWAY | -0.91 | 0.605 | 0.74 |
|  |  | CTLA4 PATHWAY | -0.90 | 0.607 | 0.74 |
|  |  | GH PATHWAY | -0.88 | 0.609 | 0.72 |
|  |  | D4GDI PATHWAY | -0.97 | 0.615 | 0.71 |
|  |  | KERATINOCYTE PATHWAY | -0.90 | 0.617 | 0.75 |
|  |  | IL7 PATHWAY | -0.93 | 0.623 | 0.72 |
|  |  | RACCYCD PATHWAY | -0.86 | 0.625 | 0.73 |
|  |  | EGFR SMRTE PATHWAY | -0.87 | 0.631 | 0.72 |
|  |  | CARM ER PATHWAY | -0.81 | 0.634 | 0.77 |
|  |  | LEPTIN PATHWAY | -0.82 | 0.684 | 0.77 |
|  |  | TNFR1 PATHWAY | -0.82 | 0.689 | 0.77 |
|  |  | ACTINY PATHWAY | -0.77 | 0.701 | 0.82 |
|  |  | GRANULOCYTES PATHWAY | -0.68 | 0.705 | 0.89 |
|  |  | TFF PATHWAY | -0.78 | 0.707 | 0.81 |
|  |  | TOLL PATHWAY | -0.71 | 0.709 | 0.86 |
|  |  | DNAFRAGMENT PATHWAY | -0.83 | 0.711 | 0.77 |
|  |  | MITOCHONDRIA PATHWAY | -0.87 | 0.712 | 0.72 |
|  |  | BARRESTIN PATHWAY | -0.86 | 0.712 | 0.73 |
|  |  | BCELLSURVIVAL PATHWAY | -0.91 | 0.713 | 0.74 |
|  |  | CDMAC PATHWAY | -0.89 | 0.713 | 0.72 |
|  |  | DEATH PATHWAY | -0.79 | 0.717 | 0.81 |
|  |  | GSK3 PATHWAY | -0.92 | 0.719 | 0.73 |
|  |  | SRCRPTP PATHWAY | -0.90 | 0.719 | 0.73 |
|  |  | IGF1MTOR PATHWAY | -0.72 | 0.735 | 0.86 |
|  |  | AMI PATHWAY | -0.82 | 0.740 | 0.77 |
|  |  | IL2RB PATHWAY | -0.77 | 0.740 | 0.82 |
|  |  | IL1R PATHWAY | -0.63 | 0.789 | 0.91 |
|  |  | CASPASE PATHWAY | -0.68 | 0.789 | 0.88 |
|  |  | IL4 PATHWAY | -0.64 | 0.790 | 0.90 |
|  |  | GABA PATHWAY | -0.74 | 0.796 | 0.84 |
|  |  | HIVNEF PATHWAY | -0.81 | 0.802 | 0.77 |
|  |  | TRKA PATHWAY | -0.65 | 0.820 | 0.90 |
|  |  | SARS PATHWAY | -0.81 | 0.824 | 0.77 |
|  |  | WNT PATHWAY | -0.77 | 0.824 | 0.81 |
|  |  | RNA PATHWAY | -0.66 | 0.829 | 0.89 |
|  |  | FREE PATHWAY | -0.52 | 0.829 | 0.98 |
|  |  | RANKL PATHWAY | -0.63 | 0.908 | 0.91 |
|  |  | EIF4 PATHWAY | -0.52 | 0.909 | 0.99 |
|  |  | PROTEASOME PATHWAY | -0.56 | 0.911 | 0.97 |
|  |  | INTRINSIC PATHWAY | -0.67 | 0.911 | 0.89 |
|  |  | EGF PATHWAY | -0.70 | 0.913 | 0.86 |
|  |  |  |  |  |  |
| KEGG | TST | ASTHMA | 1.63 | 0.000 | 0.65 |
|  |  | NON HOMOLOGOUS END JOINING | 1.36 | 0.000 | 1.00 |
|  |  | NEUROACTIVE LIGAND RECEPTOR INTERACTION | 1.35 | 0.000 | 1.00 |
|  |  | TAURINE AND HYPOTAURINE METABOLISM | 1.47 | 0.110 | 1.00 |
|  |  | LYSINE DEGRADATION | 1.03 | 0.110 | 1.00 |
|  |  | ABC TRANSPORTERS | 1.20 | 0.111 | 1.00 |
|  |  | HISTIDINE METABOLISM | 1.26 | 0.114 | 1.00 |
|  |  | AUTOIMMUNE THYROID DISEASE | 1.37 | 0.114 | 1.00 |
|  |  | GLYCINE SERINE AND THREONINE METABOLISM | 1.32 | 0.115 | 1.00 |
|  |  | GLYCOSPHINGOLIPID BIOSYNTHESIS GANGLIO SERIES | 1.11 | 0.193 | 1.00 |
|  |  | RIBOFLAVIN METABOLISM | 1.17 | 0.201 | 1.00 |
|  |  | MATURITY ONSET DIABETES OF THE YOUNG | 1.27 | 0.205 | 1.00 |
|  |  | PRIMARY BILE ACID BIOSYNTHESIS | 1.08 | 0.226 | 1.00 |
|  |  | STEROID HORMONE BIOSYNTHESIS | 1.24 | 0.230 | 1.00 |
|  |  | RNA DEGRADATION | 1.12 | 0.232 | 1.00 |
|  |  | NITROGEN METABOLISM | 1.13 | 0.291 | 1.00 |
|  |  | TYROSINE METABOLISM | 1.20 | 0.295 | 1.00 |
|  |  | NICOTINATE AND NICOTINAMIDE METABOLISM | 1.08 | 0.302 | 1.00 |
|  |  | PENTOSE AND GLUCURONATE INTERCONVERSIONS | 1.22 | 0.305 | 1.00 |
|  |  | PHENYLALANINE METABOLISM | 1.08 | 0.308 | 1.00 |
|  |  | GLYCEROPHOSPHOLIPID METABOLISM | 1.04 | 0.308 | 1.00 |
|  |  | PORPHYRIN AND CHLOROPHYLL METABOLISM | 0.93 | 0.384 | 1.00 |
|  |  | PANTOTHENATE AND COA BIOSYNTHESIS | 1.07 | 0.392 | 1.00 |
|  |  | GLYCOSAMINOGLYCAN BIOSYNTHESIS CHONDROITIN SULFATE | 0.97 | 0.394 | 1.00 |
|  |  | STEROID BIOSYNTHESIS | 0.86 | 0.403 | 1.00 |
|  |  | ALANINE ASPARTATE AND GLUTAMATE METABOLISM | 0.93 | 0.407 | 1.00 |
|  |  | RETINOL METABOLISM | 1.08 | 0.418 | 1.00 |
|  |  | METABOLISM OF XENOBIOTICS BY CYTOCHROME P450 | 1.00 | 0.424 | 1.00 |
|  |  | DRUG METABOLISM OTHER ENZYMES | 0.97 | 0.425 | 1.00 |
|  |  | FOLATE BIOSYNTHESIS | 0.96 | 0.430 | 1.00 |
|  |  | GLYCOSAMINOGLYCAN BIOSYNTHESIS HEPARAN SULFATE | 1.08 | 0.433 | 1.00 |
|  |  | LYSOSOME | 0.74 | 0.478 | 1.00 |
|  |  | ONE CARBON POOL BY FOLATE | 0.97 | 0.489 | 1.00 |
|  |  | TRYPTOPHAN METABOLISM | 0.81 | 0.498 | 1.00 |
|  |  | GLYOXYLATE AND DICARBOXYLATE METABOLISM | 0.86 | 0.509 | 1.00 |
|  |  | BIOSYNTHESIS OF UNSATURATED FATTY ACIDS | 0.90 | 0.510 | 1.00 |
|  |  | SNARE INTERACTIONS IN VESICULAR TRANSPORT | 0.76 | 0.575 | 1.00 |
|  |  | RIG I LIKE RECEPTOR SIGNALING PATHWAY | 0.85 | 0.590 | 1.00 |
|  |  | VALINE LEUCINE AND ISOLEUCINE DEGRADATION | 0.71 | 0.613 | 1.00 |
|  |  | LIMONENE AND PINENE DEGRADATION | 0.89 | 0.616 | 1.00 |
|  |  | DRUG METABOLISM CYTOCHROME P450 | 0.90 | 0.621 | 1.00 |
|  |  | RNA POLYMERASE | 0.90 | 0.635 | 1.00 |
|  |  | PYRUVATE METABOLISM | 0.66 | 0.682 | 1.00 |
|  |  | ALLOGRAFT REJECTION | 0.75 | 0.684 | 1.00 |
|  |  | CYTOSOLIC DNA SENSING PATHWAY | 0.78 | 0.703 | 1.00 |
|  |  | BUTANOATE METABOLISM | 0.69 | 0.704 | 1.00 |
|  |  | AMINOACYL TRNA BIOSYNTHESIS | 0.71 | 0.708 | 1.00 |
|  |  | GRAFT VERSUS HOST DISEASE | 0.82 | 0.718 | 1.00 |
|  |  | BASAL TRANSCRIPTION FACTORS | 0.79 | 0.719 | 1.00 |
|  |  | GLYCOSYLPHOSPHATIDYLINOSITOL GPI ANCHOR BIOSYNTHESIS | 0.79 | 0.724 | 1.00 |
|  |  | PEROXISOME | 0.80 | 0.730 | 1.00 |
|  |  | N GLYCAN BIOSYNTHESIS | 0.68 | 0.731 | 1.00 |
|  |  | TYPE II DIABETES MELLITUS | 0.83 | 0.786 | 1.00 |
|  |  | SELENOAMINO ACID METABOLISM | 0.83 | 0.801 | 1.00 |
|  |  | NUCLEOTIDE EXCISION REPAIR | 0.77 | 0.803 | 1.00 |
|  |  | ALPHA LINOLENIC ACID METABOLISM | 0.61 | 0.803 | 1.00 |
|  |  | JAK STAT SIGNALING PATHWAY | 0.79 | 0.805 | 1.00 |
|  |  | ACUTE MYELOID LEUKEMIA | 0.83 | 0.812 | 1.00 |
|  |  | CIRCADIAN RHYTHM MAMMAL | 0.67 | 0.814 | 1.00 |
|  |  | PROTEIN EXPORT | 0.45 | 0.818 | 1.00 |
|  |  | PROPANOATE METABOLISM | 0.54 | 0.825 | 1.00 |
|  |  | TASTE TRANSDUCTION | 0.77 | 0.829 | 1.00 |
|  |  | OTHER GLYCAN DEGRADATION | 0.54 | 0.906 | 1.00 |
|  |  | PRIMARY IMMUNODEFICIENCY | 0.74 | 0.906 | 1.00 |
|  |  | THYROID CANCER | 0.55 | 0.910 | 1.00 |
|  |  | BETA ALANINE METABOLISM | 0.51 | 0.911 | 1.00 |
|  |  | PROTEASOME | 0.30 | 0.911 | 1.00 |
|  |  | REGULATION OF AUTOPHAGY | 0.48 | 0.912 | 1.00 |
|  |  | GLYCEROLIPID METABOLISM | 0.63 | 0.914 | 1.00 |
|  |  |  |  |  |  |
|  | TT | GAP JUNCTION | -1.45 | 0.000 | 1.00 |
|  |  | OOCYTE MEIOSIS | -1.37 | 0.000 | 1.00 |
|  |  | VASCULAR SMOOTH MUSCLE CONTRACTION | -1.35 | 0.000 | 1.00 |
|  |  | O GLYCAN BIOSYNTHESIS | -1.32 | 0.000 | 1.00 |
|  |  | CALCIUM SIGNALING PATHWAY | -1.32 | 0.000 | 1.00 |
|  |  | MELANOGENESIS | -1.28 | 0.000 | 1.00 |
|  |  | GLIOMA | -1.28 | 0.000 | 1.00 |
|  |  | MELANOMA | -1.25 | 0.000 | 1.00 |
|  |  | BLADDER CANCER | -1.24 | 0.000 | 0.98 |
|  |  | PATHWAYS IN CANCER | -1.22 | 0.000 | 0.88 |
|  |  | BASE EXCISION REPAIR | -1.22 | 0.000 | 0.86 |
|  |  | PROGESTERONE MEDIATED OOCYTE MATURATION | -1.19 | 0.000 | 0.80 |
|  |  | CHRONIC MYELOID LEUKEMIA | -1.18 | 0.000 | 0.85 |
|  |  | SMALL CELL LUNG CANCER | -1.16 | 0.000 | 0.82 |
|  |  | PURINE METABOLISM | -1.15 | 0.000 | 0.79 |
|  |  | LONG TERM POTENTIATION | -1.41 | 0.094 | 1.00 |
|  |  | RENAL CELL CARCINOMA | -1.25 | 0.095 | 1.00 |
|  |  | TGF BETA SIGNALING PATHWAY | -1.17 | 0.095 | 0.85 |
|  |  | CELL CYCLE | -1.15 | 0.095 | 0.78 |
|  |  | FRUCTOSE AND MANNOSE METABOLISM | -1.31 | 0.097 | 1.00 |
|  |  | GLUTATHIONE METABOLISM | -1.13 | 0.097 | 0.78 |
|  |  | DORSO VENTRAL AXIS FORMATION | -1.23 | 0.105 | 0.90 |
|  |  | WNT SIGNALING PATHWAY | -1.36 | 0.111 | 1.00 |
|  |  | FOCAL ADHESION | -1.36 | 0.112 | 1.00 |
|  |  | DILATED CARDIOMYOPATHY | -1.25 | 0.112 | 1.00 |
|  |  | RIBOSOME | -1.47 | 0.120 | 1.00 |
|  |  | VEGF SIGNALING PATHWAY | -1.25 | 0.185 | 1.00 |
|  |  | CARDIAC MUSCLE CONTRACTION | -1.10 | 0.185 | 0.82 |
|  |  | TIGHT JUNCTION | -1.24 | 0.189 | 0.94 |
|  |  | B CELL RECEPTOR SIGNALING PATHWAY | -1.23 | 0.189 | 0.92 |
|  |  | LEUKOCYTE TRANSENDOTHELIAL MIGRATION | -1.16 | 0.189 | 0.79 |
|  |  | LONG TERM DEPRESSION | -1.20 | 0.196 | 0.82 |
|  |  | ECM RECEPTOR INTERACTION | -1.43 | 0.207 | 1.00 |
|  |  | MAPK SIGNALING PATHWAY | -1.32 | 0.207 | 1.00 |
|  |  | HYPERTROPHIC CARDIOMYOPATHY HCM | -1.20 | 0.207 | 0.84 |
|  |  | EPITHELIAL CELL SIGNALING IN HELICOBACTER PYLORI INFECTION | -1.15 | 0.207 | 0.77 |
|  |  | ARRHYTHMOGENIC RIGHT VENTRICULAR CARDIOMYOPATHY ARVC | -1.31 | 0.210 | 1.00 |
|  |  | GNRH SIGNALING PATHWAY | -1.24 | 0.210 | 1.00 |
|  |  | VASOPRESSIN REGULATED WATER REABSORPTION | -1.16 | 0.211 | 0.81 |
|  |  | VIRAL MYOCARDITIS | -1.22 | 0.289 | 0.84 |
|  |  | T CELL RECEPTOR SIGNALING PATHWAY | -1.10 | 0.294 | 0.84 |
|  |  | AMYOTROPHIC LATERAL SCLEROSIS ALS | -1.17 | 0.296 | 0.86 |
|  |  | HEDGEHOG SIGNALING PATHWAY | -1.10 | 0.297 | 0.80 |
|  |  | REGULATION OF ACTIN CYTOSKELETON | -1.20 | 0.302 | 0.85 |
|  |  | CHEMOKINE SIGNALING PATHWAY | -1.01 | 0.305 | 0.87 |
|  |  | PENTOSE PHOSPHATE PATHWAY | -1.05 | 0.305 | 0.82 |
|  |  | DNA REPLICATION | -1.08 | 0.308 | 0.82 |
|  |  | PANCREATIC CANCER | -1.09 | 0.312 | 0.82 |
|  |  | FC GAMMA R MEDIATED PHAGOCYTOSIS | -1.06 | 0.312 | 0.85 |
|  |  | VALINE LEUCINE AND ISOLEUCINE BIOSYNTHESIS | -1.14 | 0.316 | 0.77 |
|  |  | INSULIN SIGNALING PATHWAY | -1.07 | 0.316 | 0.84 |
|  |  | PARKINSONS DISEASE | -1.01 | 0.389 | 0.88 |
|  |  | HUNTINGTONS DISEASE | -1.00 | 0.389 | 0.88 |
|  |  | PROXIMAL TUBULE BICARBONATE RECLAMATION | -1.06 | 0.391 | 0.85 |
|  |  | AXON GUIDANCE | -1.01 | 0.391 | 0.89 |
|  |  | GLYCOSAMINOGLYCAN BIOSYNTHESIS KERATAN SULFATE | -1.05 | 0.400 | 0.83 |
|  |  | OLFACTORY TRANSDUCTION | -0.92 | 0.406 | 0.84 |
|  |  | SPLICEOSOME | -0.95 | 0.409 | 0.82 |
|  |  | COMPLEMENT AND COAGULATION CASCADES | -0.81 | 0.411 | 0.87 |
|  |  | BASAL CELL CARCINOMA | -0.94 | 0.417 | 0.83 |
|  |  | CYSTEINE AND METHIONINE METABOLISM | -0.95 | 0.421 | 0.83 |
|  |  | NON SMALL CELL LUNG CANCER | -0.97 | 0.424 | 0.85 |
|  |  | PHOSPHATIDYLINOSITOL SIGNALING SYSTEM | -0.99 | 0.430 | 0.86 |
|  |  | ARACHIDONIC ACID METABOLISM | -1.03 | 0.494 | 0.84 |
|  |  | NOTCH SIGNALING PATHWAY | -0.99 | 0.497 | 0.88 |
|  |  | INOSITOL PHOSPHATE METABOLISM | -0.99 | 0.503 | 0.85 |
|  |  | CITRATE CYCLE TCA CYCLE | -1.04 | 0.504 | 0.85 |
|  |  | FC EPSILON RI SIGNALING PATHWAY | -0.98 | 0.510 | 0.86 |
|  |  | OXIDATIVE PHOSPHORYLATION | -0.90 | 0.510 | 0.85 |
|  |  | NEUROTROPHIN SIGNALING PATHWAY | -1.06 | 0.513 | 0.84 |
|  |  | UBIQUITIN MEDIATED PROTEOLYSIS | -1.00 | 0.513 | 0.88 |
|  |  | P53 SIGNALING PATHWAY | -0.99 | 0.513 | 0.87 |
|  |  | ERBB SIGNALING PATHWAY | -0.96 | 0.513 | 0.84 |
|  |  | PATHOGENIC ESCHERICHIA COLI INFECTION | -0.97 | 0.515 | 0.85 |
|  |  | HOMOLOGOUS RECOMBINATION | -0.96 | 0.519 | 0.83 |
|  |  | MTOR SIGNALING PATHWAY | -0.97 | 0.525 | 0.87 |
|  |  | ASCORBATE AND ALDARATE METABOLISM | -0.87 | 0.602 | 0.88 |
|  |  | SULFUR METABOLISM | -0.80 | 0.604 | 0.88 |
|  |  | MISMATCH REPAIR | -0.74 | 0.604 | 0.92 |
|  |  | COLORECTAL CANCER | -0.93 | 0.613 | 0.83 |
|  |  | ADHERENS JUNCTION | -0.96 | 0.614 | 0.84 |
|  |  | PPAR SIGNALING PATHWAY | -0.71 | 0.617 | 0.93 |
|  |  | PRION DISEASES | -0.89 | 0.619 | 0.86 |
|  |  | LEISHMANIA INFECTION | -0.93 | 0.621 | 0.84 |
|  |  | GLYCOLYSIS GLUCONEOGENESIS | -0.87 | 0.623 | 0.89 |
|  |  | CYTOKINE CYTOKINE RECEPTOR INTERACTION | -0.87 | 0.625 | 0.89 |
|  |  | NATURAL KILLER CELL MEDIATED CYTOTOXICITY | -0.81 | 0.627 | 0.87 |
|  |  | INTESTINAL IMMUNE NETWORK FOR IGA PRODUCTION | -0.83 | 0.631 | 0.90 |
|  |  | SYSTEMIC LUPUS ERYTHEMATOSUS | -0.84 | 0.633 | 0.91 |
|  |  | ENDOCYTOSIS | -0.97 | 0.639 | 0.86 |
|  |  | LINOLEIC ACID METABOLISM | -0.82 | 0.673 | 0.89 |
|  |  | GALACTOSE METABOLISM | -0.81 | 0.680 | 0.88 |
|  |  | GLYCOSPHINGOLIPID BIOSYNTHESIS GLOBO SERIES | -0.79 | 0.693 | 0.88 |
|  |  | ADIPOCYTOKINE SIGNALING PATHWAY | -0.90 | 0.696 | 0.85 |
|  |  | PYRIMIDINE METABOLISM | -0.77 | 0.701 | 0.89 |
|  |  | GLYCOSAMINOGLYCAN DEGRADATION | -0.82 | 0.704 | 0.89 |
|  |  | VIBRIO CHOLERAE INFECTION | -0.96 | 0.714 | 0.85 |
|  |  | SPHINGOLIPID METABOLISM | -0.83 | 0.714 | 0.90 |
|  |  | HEMATOPOIETIC CELL LINEAGE | -0.78 | 0.714 | 0.89 |
|  |  | TOLL LIKE RECEPTOR SIGNALING PATHWAY | -0.73 | 0.724 | 0.93 |
|  |  | ALZHEIMERS DISEASE | -0.83 | 0.726 | 0.90 |
|  |  | CELL ADHESION MOLECULES CAMS | -0.92 | 0.734 | 0.85 |
|  |  | APOPTOSIS | -0.84 | 0.734 | 0.91 |
|  |  | FATTY ACID METABOLISM | -0.49 | 0.785 | 0.99 |
|  |  | ARGININE AND PROLINE METABOLISM | -0.84 | 0.801 | 0.92 |
|  |  | ALDOSTERONE REGULATED SODIUM REABSORPTION | -0.82 | 0.809 | 0.90 |
|  |  | TERPENOID BACKBONE BIOSYNTHESIS | -0.61 | 0.811 | 0.96 |
|  |  | STARCH AND SUCROSE METABOLISM | -0.80 | 0.817 | 0.87 |
|  |  | RENIN ANGIOTENSIN SYSTEM | -0.72 | 0.817 | 0.93 |
|  |  | ANTIGEN PROCESSING AND PRESENTATION | -0.61 | 0.821 | 0.96 |
|  |  | PROSTATE CANCER | -0.94 | 0.826 | 0.83 |
|  |  | ETHER LIPID METABOLISM | -0.68 | 0.909 | 0.95 |
|  |  | AMINO SUGAR AND NUCLEOTIDE SUGAR METABOLISM | -0.64 | 0.911 | 0.96 |
|  |  | GLYCOSPHINGOLIPID BIOSYNTHESIS LACTO AND NEOLACTO SERIES | -0.64 | 0.911 | 0.98 |
|  |  | NOD LIKE RECEPTOR SIGNALING PATHWAY | -0.64 | 0.913 | 0.97 |
|  |  | TYPE I DIABETES MELLITUS | -0.61 | 0.914 | 0.97 |
|  |  | ENDOMETRIAL CANCER | -0.60 | 0.915 | 0.95 |
|  |  |  |  |  |  |
| LIVER CANCER SIGNATURES | TST | HOSHIDA S3 | 1.09 | 0.210 | 0.47 |
|  |  | CHIANG LIVER CANCER SUBCLASS POLYSOMY7 | 1.09 | 0.244 | 0.88 |
|  |  | BOYAULT LIVER CANCER SUBCLASS G12 | 0.82 | 0.791 | 0.75 |
|  |  |  |  |  |  |
|  | TT | KUROKAWA LIVER CANCER EARLY RECURRENCE | -1.67 | 0.000 | 0.05 |
|  |  | LEE LIVER CANCER HEPATOBLAST | -1.17 | 0.000 | 0.48 |
|  |  | YE LIVER CANCER INTRAHEPATIC METS | -1.20 | 0.075 | 0.51 |
|  |  | BOYAULT LIVER CANCER SUBCLASS G56 | -1.36 | 0.077 | 0.33 |
|  |  | CHIANG LIVER CANCER SUBCLASS CTNNB1 | -1.14 | 0.079 | 0.40 |
|  |  | WOO LIVER CANCER RECURRENCE | -1.35 | 0.088 | 0.26 |
|  |  | WOO LIVER CANCER CHOLANGIOCA LIKE UP | -1.22 | 0.088 | 0.59 |
|  |  | HOSHIDA S1 | -1.16 | 0.088 | 0.46 |
|  |  | CHIANG LIVER CANCER SUBCLASS PROLIFERATION | -1.08 | 0.118 | 0.48 |
|  |  | YOSHIOKA LIVER CANCER EARLY RECURRENCE | -1.11 | 0.212 | 0.49 |
|  |  | YAMASHITA LIVER CANCER EPCAM | -1.22 | 0.295 | 0.52 |
|  |  | BOYAULT LIVER CANCER SUBCLASS G3 | -1.15 | 0.311 | 0.44 |
|  |  | WANG LIVER CANCER RECURRENCE | -1.09 | 0.397 | 0.51 |
|  |  | COULOUARN LIVER CANCER TGF BETA LATE VS EARLY | -0.97 | 0.406 | 0.67 |
|  |  | CK19SIGNATURE FDR010 | -1.01 | 0.410 | 0.68 |
|  |  | LEE LIVER CANCER POOR SURVIVAL | -1.15 | 0.414 | 0.43 |
|  |  | CAIRO LIVER CANCER HEPATOBLAST | -1.09 | 0.489 | 0.51 |
|  |  | ROESSLER LIVER CANCER METASTASIS UP | -0.97 | 0.529 | 0.70 |
|  |  | VI SIGNATURE FDR0 ABSDIFF100 | -0.82 | 0.687 | 0.81 |
|  |  | NOVAK MET | -0.84 | 0.789 | 0.84 |
|  |  | HOSHIDA S2 | -0.72 | 0.789 | 0.94 |
|  |  | CHIANG LIVER CANCER SUBCLASS INTERFERON | -0.61 | 0.820 | 0.96 |
